# Supplementary material for: Evaluating possible intended and unintended consequences of the implementation of alcohol minimum unit pricing (MUP) in Scotland: a natural experiment protocol
Source: BMJ Open. 2019 Jun 20;9(6):e028482. doi: 10.1136/bmjopen-2018-028482 (PMC6596978; doi:10.1136/bmjopen-2018-028482)
Supplement: Author's manuscript [file bmjopen-2018-028482.draft_revisions.pdf]

BMJ Open

BMJ Open is committed to open peer review. As part of this commitment we make the peer review history of every article we publish publicly available.

When an article is published we post the peer reviewers' comments and the authors' responses online. We also post the versions of the paper that were used during peer review. These are the versions that the peer review comments apply to.

The versions of the paper that follow are the versions that were submitted during the peer review process. They are not the versions of record or the final published versions. They should not be cited or distributed as the published version of this manuscript.

BMJ Open is an open access journal and the full, final, typeset and author-corrected version of record of the manuscript is available on our site with no access controls, subscription charges or pay-per-view fees (http://bmjopen.bmj.com).

If you have any questions on BMJ Open's open peer review process please email info.bmjopen@bmj.com

BMJ Open

Evaluating possible intended and unintended consequences of the implementation of minimum unit pricing (MUP) in Scotland: a natural experiment

Journal:	*BMJ Open*
Manuscript ID	bmjopen-2018-028482
Article Type:	Protocol
Date Submitted by the Author:	10-Dec-2018

Complete List of Authors:	Katikireddi, Srinivasa; University of Glasgow School of Life Sciences, MRC/CSO Social & Public Health Sciences Unit<br>Beeston, Clare; NHS Health Scotland,<br>Millard, Andrew; University of Glasgow, MRC/CSO Social and Public Health Sciences Unit<br>Forsyth, Ross; University of Glasgow School of Life Sciences, MRC/CSO Social & Public Health Sciences Unit<br>Deluca, Paolo; King's College London, Addictions<br>Drummond, Colin; Kings College London, Institute of Psychiatry<br>Eadie, Douglas; Institute for Social Marketing, School of Health Sciences<br>Graham, Lesley; ISD Scotland<br>Hilton , Shona; University of Glasgow, MRC/CSO Social & Public Health Sciences Unit<br>Ludbrook, Anne; University of Aberdeen, Health Economics Research Unit<br>McCartney, Gerry; NHS Health Scotland<br>Phillips, Thomas; University of Hull, Faculty of Health Sciences Institute for Clinical and Applied Health Research (ICAHR)<br>Stead, Martine; University of Stirling and the Open University, Institute for Social Marketing<br>Ford, Allison; University of Stirling, Institute for Social Marketing<br>Bond, Lyndal; Australian Health Policy Collaboration, Victoria University,<br>Leyland, Alastair; MRC/CSO Social and Public Health Sciences Unit, University of Glasgow,

Keywords:	ACCIDENT & EMERGENCY MEDICINE, MENTAL HEALTH, Substance misuse < PSYCHIATRY

SCHOLARONE™
Manuscripts

MUP protocol paper v6 07.12.18

Evaluating possible intended and unintended consequences of the implementation of minimum unit pricing (MUP) in Scotland: a natural experiment

Srinivasa Vittal Katikireddi: Vittal.Katikireddi@glasgow.ac.uk [1, 2]

Clare Beeston: clare.beeston@nhs.net [3]

Andrew Denis Millard: Andrew.millard@glasgow.ac.uk [1]

Ross Forsyth: Ross.Forsyth@glasgow.ac.uk [1]

Paolo Deluca: paolo.deluca@kcl.ac.uk [4]

Colin Drummond: colin.drummond@kcl.ac.uk [4]

Douglas Eadie: douglas.eadie@stir.ac.uk [5]

Lesley Graham: lesley.graham@nhs.net [6]

Shona Hilton: Shona.Hilton@glasgow.ac.uk [1]

Anne Ludbrook: a.ludbrook@abdn.ac.uk [7]

Gerry McCartney: gmccartney@nhs.net [3]

Thomas Phillips: Thomas.Phillips@hull.ac.uk [4, 8]

Martine Stead: martine.stead@stir.ac.uk [5]

Allison Ford: a.j.ford@stir.ac.uk [5]

Lyndal Bond: Lyndal.Bond@vu.edu.au [9]

Alastair H Leyland: Alastair.Leyland@glasgow.ac.uk [1]

[1] MRC/CSO Social and Public Health Sciences Unit, University of Glasgow

[2] Department of Public Health and Health Policy, NHS Lothian

[3] NHS Health Scotland

[4] National Addiction Centre, Institute of Psychiatry, Psychology and Neuroscience, King's College London

[5] Institute for Social Marketing, University of Stirling

[6] Information Services Division, NHS National Services Scotland

[7] Health Economics Research Unit, University of Aberdeen

[8] Institute for Clinical and Applied Health Research (ICAHR), University of Hull

[9] Australian Health Policy Collaboration, Victoria University, Australia

Correspondence to

Dr Andrew D. Millard: Andrew.millard@glasgow.ac.uk

For peer review only - http://bmjopen.bmj.com/site/about/guidelines.xhtml

MUP protocol paper v6 07.12.18

Keywords: alcohol, pricing, policy, evaluation

Abstract

Introduction

Scotland is the first country to carry out a national implementation of Minimum Unit Pricing (MUP) for alcohol. MUP aims to reduce alcohol-related harms, which are high in Scotland compared to Western Europe, and to improve health equalities. MUP is a minimum retail price per unit of alcohol. That approach targets high-risk alcohol users. This work is key to a wider evaluation that will determine whether MUP continues. There are three study components.

Methods and Analysis

Component 1 sampled an estimated 2800 interviewees at a baseline and each of two follow ups from four Emergency Departments in Scotland and Northern England. Research nurses administered a standardised survey to assess alcohol consumption and the proportion of attendances that were alcohol-related.

Component 2 covered six Sexual Health Clinics with similar timings and country allocation. A self-completion survey gathered information on potential unintended effects of MUP on alcohol source and drug use.

Using a natural experiment design and repeated cross-sectional audit, difference in difference between Scotland (intervention) and North England (control) will be tested for outcomes using regression adjusting for differences at baseline. Differential impacts by age, gender and socioeconomic position will be investigated.

Component 3 used focus groups with young people and heavy drinkers and interviews with stakeholders before and after MUP implementation. The focus groups will allow exploration of attitudes, experiences and behaviours, and the potential mechanisms by which impacts arise. The interviews will help characterise the implementation process.

Ethics and Dissemination

Study components 1 and 2 have been ethically approved by the NHS, and component 3 by the University of Stirling. Dissemination plans include peer-reviewed journal articles, presentations, policy maker briefings and, in view of high public interest and the high political profile of this flagship policy, communication with the public via media engagement and plain language summaries.

300/300 words

Main paper 5391 words

For peer review only - http://bmjopen.bmj.com/site/about/guidelines.xhtml

Strengths and limitations of this study

- This evaluation adopts multiple methods to help establish whether MUP has caused changes in alcohol-related attendances using a natural experiment design, which is the most appropriate for this topic as a randomized controlled trial would not be feasible.
- Our study exploits divergences in Scottish and English alcohol policy to evaluate the effectiveness of an all-beverage MUP for the first time, and evaluates both positive and possible negative impacts of minimum unit pricing of alcohol; such negative impacts might include use of other sources of alcohol, other substances, or reduction in money available for essentials.
- The Northern English control group is likely to be comparable to Scotland because of geographical proximity and similar levels of deprivation, but we also assess the external validity of our sample by reference to routine data on attendances at Emergency Departments and Sexual Health Clinics.
- There is the potential to follow up individuals through longer term data linkage, for which we will obtain respondents' permission, thus adding a cohort dimension to the initial cross-sectional approach.
- The main limitation is that the non-randomised design risks selection bias, for example in the differential selection of intoxicated attendees for interview.

For peer review only

Introduction

Minimum unit pricing (MUP) of alcohol is a novel public health policy that aims to reduce alcohol-related harms across the population. In May 2012, Scotland became the first country to pass legislation to introduce MUP without reference to beverage type, a politically high profile measure,[1-3] although some Canadian provinces have introduced beverage-specific MUP, as further described below. This paper describes the protocol for three associated studies that collect primary data to evaluate the impact of the policy in Scotland. After delays following legal challenges,[4 5] MUP legislation was implemented in May 2018 but it is due to cease in six years unless shown to be effective, under a 'sunset clause'. These studies are a key part of the evaluation planned by NHS Health Scotland, which will report to Scottish Ministers and the Scottish Parliament. The evaluation includes analyses by others of routinely collected administrative data.[6]

The overall aim of the study is to investigate the impacts of MUP on acute and chronic health harms (including by deprivation and gender and age subgroups). The study will also determine the extent to which specific unintended consequences occur. The evidence will be a key input to the decision whether the policy will continue.

Alcohol consumption is a major determinant of population health globally,[7] and the level of alcohol-related harm in Scotland is high compared to the rest of Western Europe.[8] Alcohol is associated with over 200 medical conditions including an increased risk of liver disease, heart disease, unintended pregnancy, sexually transmitted infections, some cancers and accidental injuries.[9-14] Its impact extends beyond the individual, with adverse effects on families, communities and the wider economy.[15 16] Furthermore, alcohol is known to be a major contributor to socioeconomic inequalities in health in the UK and elsewhere.[17-20] From a public health perspective, alcohol therefore has considerable potential as a modifiable risk factor to be addressed to help reduce health inequalities.[21-23]

A consistent body of international evidence demonstrates a negative relationship between price, consumption and alcohol-related harms.[24-28] The World Health Organization has identified changing the price of alcohol, and controls on promotion and availability, as key methods for addressing alcohol-related health harms.[29 30 31] Across much of the world, the best established mechanism for influencing price has been taxation through alcohol duty, although in many countries, including the UK, the primary purpose is revenue-raising for central government rather than achieving public health goals.[32] As a result of this secondary consideration of public health, alcohol duty may be poorly designed to address alcohol-related harms since duty typically varies by product in a manner not consistent with the associated harms arising from the product's consumption. At the time of writing tax on a litre of 7.5% ABV cider is 40 pence, on a litre of wine of the same strength tax would be 289 pence.[33]

A potentially complementary approach to increasing alcohol duty is the introduction of a floor price, such as MUP, below which alcohol should not be retailed.[34 35] MUP sets a minimum price per UK unit (eight grams) of pure alcohol. Epidemiological studies have found that drinkers at the greatest risk of harm tend to consume the cheapest (per unit) alcohol, thereby providing evidence that MUP may better target harmful drinkers in comparison to alcohol duty.[36 37] Econometric modelling studies suggest that MUP will result in a greater reduction in health harms compared to an equivalent rise in taxation under the UK's current system of

For peer review only - http://bmjopen.bmj.com/site/about/guidelines.xhtml

calculating alcohol duty.[22 23 38-40] In addition, setting a floor price minimises the potential for 'trading down' to cheaper drinks, given that alcoholic drinks below the floor price would no longer be legally available. This is particularly relevant since previously retailers have opted not to pass on alcohol duty increases to consumers, as indicated by the existence of below-cost products.[41]

Comparable interventions to MUP have been introduced elsewhere. The best known example is in Canada, where some Canadian provinces introduced a minimum price for selling specific beverages (also referred to as 'reference pricing') within the context of government-operated monopolies.[42 43] While some authors refer to these interventions as 'minimum pricing', the nature of this policy differs in some aspects from 'minimum unit pricing' as planned in Scotland.[3 5 44] In contrast to MUP which applies a uniform minimum price per unit across all beverage types, reference pricing imposes differing minimum prices that are determined by both alcohol strength and drink type. In addition, MUP in Scotland was introduced into a competitive alcohol market at a national level, in contrast to the locally applied government-owned monopolies in which reference pricing has been introduced. It is also worth noting that reference pricing was originally introduced primarily for its revenue-raising potential and not its potential public health benefits. Important benefits of reference pricing in Canada have nevertheless been demonstrated, with reductions in alcohol consumption, alcohol-attributable hospital admissions and crime observed.[45-50] However, a study focusing on Emergency Department (ED) visits found no reduction in overall visits for alcohol-related injuries, although alcohol-related motor vehicle injuries did fall.[51] Other broadly comparable pricing interventions, such as the abolition of cheap vodka within communist Russia, have similarly been associated with reductions in alcohol-related mortality.[52]

In Scotland, qualitative research has investigated the policy process through which MUP developed, including assessing the role of commercial interests, and seeking to identify transferable lessons for public health advocacy.[3 5 44 53-58] The influence of econometric modelling has been specifically investigated.[59 60]The dominant media discourses and the roles of different policy stakeholders in articulating arguments to the public have been explored using content analysis of newspaper reporting and trends in newspaper coverage have been tracked over time.[61-64] The views of the public and heavy drinkers around MUP have also been investigated.[65-68] There remained a need to investigate the views of policy stakeholders, the public, and heavy drinkers about MUP as implemented.

Empirical research gathering primary data specifically focused on MUP in Scotland remains limited. The introduction of MUP provides a unique opportunity to use a natural experiment to evaluate public health policy, and provide new real-world evidence on the effectiveness of MUP, where most evidence to date has been from modelling studies.[69 70] A broad programme of research is being co-ordinated by NHS Health Scotland, which will report to Scottish Ministers and the Scottish Parliament between five and six years after the start of the policy. This programme includes analyses of administrative data and alcohol sales data.[71] The protocol described here complements the NHS-led work and has been funded by the National Institute for Health Research (NIHR) Public Health Research programme to collect primary data in three associated studies.

Much of the burden of alcohol-related harm, especially amongst young people, arises from acute harm following high episodic consumption ('binge drinking'). These harms are of

For peer review only - http://bmjopen.bmj.com/site/about/guidelines.xhtml

particular interest to policymakers as they may be associated with social disorder and violence. That in turn provides one justification for MUP, although the case for MUP legislation in Scotland was fought (and won) on public health benefits. Comprehensive diagnostic data on ED alcohol-related attendances that do not result in admission are currently not adequately captured in administrative health data within the UK, so require primary research.

Our work focuses on both intended and possible unintended consequences of the intervention.[72] A number of potential risks arising from the introduction of MUP have been identified by policymakers, the alcohol industry and public health professionals.[54]

1. Consumers may switch to alternative sources of alcohol not subject to MUP so that the price paid does not increase. Such sources include both legal sources: internet sales from outside Scotland, legitimate cross-border purchase for own use, and home fermentation) and illegal sources (counterfeit, and unlicensed sale of smuggled or stolen alcohol).
2. Increased alcohol-related harm could occur through substitution (e.g. to illicitly produced or industrial alcohol associated with greater toxicity) or changed drinking patterns (e.g. moving from regular drinking to binge drinking).
3. Displacement effects with reductions in alcohol-related harms potentially accompanied by increases in harms related to other substance use.
4. MUP could unfairly penalise deprived populations less able to absorb the additional financial cost and this may adversely affect access to essentials such as food and fuel.
5. MUP may have unintended effects on harmful drinkers who cannot reduce their consumption.
6. There may be adverse economic impacts on the Scottish alcohol industry retailers and/or manufacturers.

The ED and Sexual Health Clinics (SHC) studies described in this protocol will address the first four of these potential risks, although analysis of existing datasets by NHS Health Scotland will further address the potential impact on access to essential goods. A study has been commissioned from the University of Sheffield by NHS Health Scotland to assess the effects on harmful drinkers. The possible adverse economic impact on the Scottish alcohol industry will not be addressed by our project but will be monitored by the Scottish Government through the Monitoring and Evaluating Scotland's Alcohol Strategy (MESAS) studies managed by NHS Health Scotland.

It was not possible to collect sufficiently valid consumption data in the ED setting to assess alcohol consumption levels based on consumption owing to interview time constaints. Instead, we defined hazardous, harmful and dependent alcohol consumption using scores from the Fast Alcohol Screening Test (FAST).[73] A score of >=3 on the FAST indicates hazardous drinking. We are undertaking a parallel examination of The FAST screening tool to further discriminate alcohol use disorders in ED attendees. In our study MUP is theorised to reduce alcohol misuse (hazardous, harmful and dependent alcohol consumption), so reducing both acute and chronic alcohol-related harm, and therefore the numbers of alcohol-related attendances at both EDs and at SHCs. Hazardous drinkers are expected to be more likely than harmful or dependent drinkers to present with acute harms (such as accidents, injuries and assaults), while harmful/dependent drinkers are likely to present with both acute conditions and acute consequences of chronic conditions (such as pancreatitis and gastritis).

For peer review only - http://bmjopen.bmj.com/site/about/guidelines.xhtml

Therefore there may be an early impact on ED attendances relating to chronic as well as acute alcohol-related conditions.

Research questions

The more specific research questions and objectives are summarised in Table 1, with the contribution of each of the three study components stated.

Table1 Research Aims and Study Components

**Research aims (RAs)**	**Study components (C)**
RA1: To determine the impact of MUP on alcohol-related harms and drinking patterns for the overall population and by subgroups of interest (age, sex and socioeconomic position).	ED study of alcohol-related attendances (C1);<br><br>Survey of alcohol-related behaviours (consumption patterns, alcohol spend, source of alcohol, move to other substances) in SHCs (C2)
RA2: To determine the impact of MUP on non-alcohol substance use, and other unintended impacts, for the overall population and by subgroups of interest (age, sex and socioeconomic position).	Survey of alcohol-related behaviours in SHCs (C2);<br><br>Qualitative focus group study and stakeholders (C3)
RA3: To describe changes in experiences and norms towards MUP and alcohol use following the introduction of MUP by subgroups of interest (age, sex and socioeconomic position).	Qualitative focus groups with young people/heavy drinkers and interviews with stakeholders from public services (C3)

For peer review only

Methods and analysis

Component 1: Audit of alcohol-related attendances at EDs

Study design

This component is designed to quantify changes in alcohol-related attendances at ED settings as a result of MUP. It therefore focuses on answering RA1, namely:

1. What are the impacts of MUP for alcohol on alcohol-related harms and drinking patterns (using the FAST) for ED attendees and by subgroups of interest (age, sex and deprivation)?

For peer review only - http://bmjopen.bmj.com/site/about/guidelines.xhtml

2. Does the effect of MUP vary dependent on the type of alcohol-related harm:
    a. Acute alcohol-related harms vs chronic alcohol-related harms?
    b. Broad diagnostic groups (based on coding systems used in EDs?
3. Does MUP affect alcohol consumption (on the basis of the FAST[73] score and alcohol misuse over the reporting period amongst people attending EDs (FAST $\geq$3)?
4. Does the MUP intervention effect size (assessed on a variety of measures including FAST) vary at the second and third timepoints?

Lower level outcomes are set out in the section on data collection below. To answer research aim 1, a natural experiment design employing a repeated cross-sectional audit of all alcohol-related attendances at EDs in two Scottish hospitals and two North of England comparator hospitals at three time points was most appropriate to assess causality. A Randomised Controlled Trial (RCT) was not feasible. The study methods and tools were based on previous studies used to quantify the national prevalence of alcohol-related attendances in EDs in England[74] and informed by the experience of the Scottish ED Alcohol Audit, carried out across 15-20 hospitals throughout mainland Scotland between October 2005 and June 2007.[75]

Study population

People aged 16 years and over attending EDs during data collection periods were approached. The study monitored, but excluded from interview:

- those acutely physically or mentally ill such that informed consent or full participation was impossible to obtain (including gross intoxication),
- those with whom it was impossible to adequately communicate (e.g. patients unable to speak English despite the use of an interpreter where available), and
- those who left the department before an approach was made or who were deemed by the research nurses as too threatening to approach.

Reasons for not approaching were recorded to address potential selection bias.

Data collection

Baseline data collection took place in February 2018 prior to the implementation of MUP on 1st May 2018. The 2nd and 3rd waves of data collection took place approximately eight and 12 months post baseline, and five and nine months post implementation, with care taken to avoid the inclusion of specific events that are expected to be associated with increased or decreased alcohol consumption, for example local holidays were checked to avoid differences between groups. Each wave collected data over three weeks, and to maximise collection of both alcohol-related attendances and alcohol misuse, data collection occurred from 20.00 to 03.30 on Thursday to Sunday (i.e. 30 hours per week during this time period) and 09.00 to 16.30 on Monday to Wednesday (i.e. 22.5 hours per week during this time period).

For peer review only - http://bmjopen.bmj.com/site/about/guidelines.xhtml

During study data collection periods, patients were approached by a research nurse to seek permission to complete a short face-to-face interview. All eligible patients were approached, informed consent obtained, and a face-to-face interview based on a previously used patient questionnaire was administered electronically using a tablet (iPad).[76] An ED attendance database was maintained for all attendances during data collection periods to allow monitoring of patient recruitment.

The interview collected details on the following:

- basic demographic information (age; gender; ethnicity; marital, employment and housing status; postcode sector);
- attendance details (date and time, triage category, time and date of accident/injury),
- how much alcohol was consumed in the past 24 hours;
- date, time and place of last drink;
- the largest number of drinks on any one day in the previous week;
- self-reported reason for attendance, such as assault, accident etc;
- alcohol misuse in the past year (as assessed by the FAST[73]) and
- whether alcohol consumption changed over the previous 12 months.

In addition we asked whether interviewees thought their current ED attendance was related to their or someone else's drinking, and how many times they had attended any ED in the previous 12 months.

A note was made of the hospital unique identifier for the attendance if participants agreed to link their research interview data to their ED data. Research staff retrieved additional data from patient records on full postcode, triage category, discharge status and diagnoses at discharge (coded by ICD-10 or other ED coding systems in use locally). The consent procedure asked participants for separate permissions both for this and for data linkage to facilitate longitudinal follow-up. Additionally, aggregated data on age group, sex and diagnosis were sought for all attendees in the study time periods in order to allow an assessment of the representativeness of the interviewee sample.

Socioeconomic position and inequalities

Analysis by deprivation, age and sex will be undertaken to determine if the impact of MUP on alcohol-related attendances at EDs is differentially patterned. Assessment of alcohol-related attendances within the ED setting provides a broad range of deprivation, age, sex and drinking status categories. It therefore not only provides information on an important primary health outcome but also allows adequate investigation of the intervention's impact on important subgroups.

Outcome measures

The primary outcome will be the proportion of attendances that are alcohol-related, using a composite measure which defined the attendance as alcohol-related if any one (or more) of the following was true:

- patient self-reports attendance is alcohol-related;
- patient reports alcohol consumption in past 24 hours $\geq$8 units in men, $\geq$6 units in women; or

For peer review only - http://bmjopen.bmj.com/site/about/guidelines.xhtml

- patient not approached or interview terminated because too intoxicated with alcohol.

Secondary outcomes will be:

- the absolute number and proportion of alcohol-related attendances;
- alcohol misuse defined by the FAST score $\geq$3;
- mean FAST score by age, gender, and deprivation;
- prevalence of binge drinking in the past week, and
- self-reported reason for attendance.

Outcomes will be compared between intervention and control areas, adjusted for baseline attendances.

Power calculation

Based on the experience of the 24 hour audit of EDs in England,[74] and assuming that we would be able to recruit 50% of eligible ED attendees, we anticipated that the four sites (two Scotland, two England) would result in 940 recruits per week. Recruiting over 3 weeks pre- and post-implementation – giving a total sample size of 5,640 for the baseline and any one subsequent wave - would mean that we would be highly powered (>80%) to detect an effect size of $\pm$5% in the proportion of alcohol-related attendances from an estimated 30% with 95% significance. We have used a base rate of 30% informed by the 24 hour audit of EDs in England[74] and assumed a 5% decrease would be of public health importance and may be expected based on current evidence.[74] For sub-group analyses, we would have good power (>80%) to detect an effect size of 0.23 on the FAST score among those from the most deprived quintile (estimated to be 25% of attendances) and an effect size of 0.27 among those aged 18-24 years (estimated to be 15% of attendances).

Statistical analysis

We will test for differences in the outcomes between the intervention and control groups using a regression model with ED included as a fixed effect, and with individuals nested within the ED of their attendance, before and after adjustment for relevant covariates including baseline levels of alcohol-related attendance, sex, age group, and triage category. We will also attempt to determine the nature of the effect more precisely in terms of whether there is a dose-response effect according to the time since MUP was implemented (through a test of the significance of an interaction between time and intervention). We will test for interactions of the intervention with defined important covariates (including age, gender, deprivation quintile, and disease diagnostic categories) to investigate the possibility of differential intervention effects and will subsequently stratify the analyses if indicated.

Ethical arrangements

Trained nurse researchers will approach and interview participants in order to ensure informed consent is obtained from study participants. The research team has considerable expertise with appropriately considering ethical issues in studies with similar methodology previously.[74 76] We will follow standard procedures for obtaining informed consent.

For peer review only - http://bmjopen.bmj.com/site/about/guidelines.xhtml

Participants will receive information on local alcohol services and national advice telephone lines in the participant information leaflet.

Component 2: Survey of alcohol-related behaviours in sexual health services

Young adults and high-risk alcohol users are under-represented in national surveys. SHCs were selected as the setting for data collection to provide adequate inclusion of deprived groups and young people. Understanding how MUP might change alcohol misuse patterns in this group and/or increase use of other substances is considered a priority by policymakers. SHCs provide a convenience-sampling frame to monitor changes in behaviour within a high-risk population that includes adequate representation of both deprivation and young age. This component contributes to RA1 and RA2, specifically to investigate, amongst a population at high-risk of alcohol and drug-related problems:

1. changes in alcohol misuse
2. how sources of alcohol consumption have changed following MUP
3. whether MUP has impacted on the use of psychoactive substances apart from alcohol
4. whether any unintended impacts of MUP, such as changes to alcohol sources and psychoactive substance use differ across Age group, Gender, Highest educational attainment, and Employment status
5. whether any observed intervention effects vary at the second and third timepoints

Study design

A repeated cross-sectional survey of patients attending SHCs was conducted to determine changes in drinking patterns and psychoactive substance use within this population. All patients attending three SHCs in Scotland and three in North England were invited to self-complete a short questionnaire. Data collection took place over three weeks at baseline, and eight and 12 months post-baseline. Timing was similar to the ED audit.

Study population

Patients of any age attending sexual health services participating in the study during data collection periods were approached. Patients who are unable to understand English well enough to complete the questionnaire (with assistance), or who left the clinic before an approach was made, and those deemed inappropriate by clinical staff to approach were excluded from the study.

Data collection

The questionnaire was offered to attendees at reception by a trained reception staff. Participants completed the questionnaire while waiting to be seen and returned it into a confidential returns box. The trained staff were available to offer assistance where needed. The confidential and voluntary nature of the study will be emphasised. Similar patient completed questionnaires have been carried out as part of NHS Board work within NHS

For peer review only - http://bmjopen.bmj.com/site/about/guidelines.xhtml

Lothian and NHS Greater Glasgow and Clyde achieving response rates of between 50-70% (without the assistance of support staff).

Questions covered where alcohol is procured, other substances used, the FAST tool (allowing quantification of alcohol misuse and binge drinkers), and basic characteristics (age band, sex, highest educational level). The questionnaire was designed to allow completion within between two and five minutes.

Socioeconomic position and inequalities

Trained reception staff assisted self-completion if required where literacy problems or visual impairment otherwise precluded participation. Analysis will be undertaken by highest education level, age and sex to determine if behavioural response to MUP in terms of drinking, source of alcohol and displacement is differentially patterned.

Outcome measures

The primary outcome from C2, which aims to reveal unintended effects, will be the proportion of patients self-reporting psychoactive substance use other than alcohol in the last month. Secondary outcomes will be drug-specific rates of use within the last month, sources of alcohol purchase (including cross-border, internet, smuggled or moonshine), rates of higher risk drinking (as measured by FAST score $\geq$3), self-reported binge-drinking, and analyses for differential displacement of use of illicit psychoactive drugs (including age, gender, highest achieved education, problematic drinkers) *Power calculation*

We envisaged being able to recruit at least 50% of attendees at sexual health clinics at the six sites (three Scotland, three England), therefore resulting in a total of slightly over 10,000 recruits over two 3 week periods (an average of 288 per clinic per week). This would give us power of more than 80% to detect a change of $\pm$ 4% from an estimated 30% in the proportion of people using drugs.

Statistical analysis

Similar analyses to the ED study will be undertaken based on regression models with site included as a fixed effect, and the proportion taking illicit psychoactive drugs as the dependent variable for the primary outcome. We will examine whether any displacement effects are sustained between six and 12 months post implementation. We will investigate interactions with defined important covariates (including age, gender, highest achieved education, problematic drinkers) to investigate the possibility of differential intervention harms.

Ethical arrangements

To protect patient confidentiality, no identifiable personal data was collected. A research assistant was available to provide a questionnaire, answer any questions and assist those experiencing difficulty with self-completion. Given that young people are a particular focus of this study, no lower age restriction was planned. No separate written consent was planned as completion of the questionnaire was considered to constitute implied consent.

For peer review only - http://bmjopen.bmj.com/site/about/guidelines.xhtml

MUP protocol paper v6 07.12.18

Component 3: Qualitative study of young people & heavy drinkers

The aim of this study component is to provide an in-depth understanding of how MUP is affecting key sub groups within the Scottish population. Understanding the experience of those exposed to the intervention will allow further exploration of potential mechanisms that result in unanticipated benefits and harms and which may affect groups to different degrees. This study contributes specifically to RAs 2 & 3 by exploring participants' expectations and and experiences of the policy's impact including unintended consequences, both personally and on family, friends and wider community, and by comparing narratives between the different sample groups: age, gender and socioeconomic position.

.

Research design and data collection

Focus groups and interviews were carried out in January to April 2018 and repeated in September/October 2018 in three communities in Scotland; an affluent urban community, and two deprived urban communities. Participants were sought from residents located within the catchment areas served by the two Scottish hospitals participating in the ED study component. Postcode deprivation (Carstairs scores) were used to define study communities in each area which match the socioeconomic comparators of interest (deprived and affluent).

1) Focus groups were conducted with those sub-groups who may be particularly affected by MUP: young people and heavy drinkers (previously categorised using the AUDIT C tool, a brief 3-item alcohol screening tool designed to identify hazardous an harmful drinking patterns) living in both deprived and affluent communities.. Group participants will be recruited by independent market researchers using door-to-door and street intercept techniques. Recruitment will be facilitated by the use of a recruitment questionnaire containing AUDIT-C screening questions. The discussions covered: social norms and attitudes; alcohol displacement behaviours; changing patterns in drinking and purchasing habits.

2) Individual semi-structured interviews with 15-18 professional stakeholders positioned to observe immediate social, health and economic impacts of MUP were conducted prior to the focus groups at each wave. As well as addressing the main research objectives, data from these interviews will help characterise the implementation process within each study community from differing professional perspectives and will explore adequacy of implementation and any difficulties experienced. In addition, these data will provide valuable contextual information for informing the focus group topic guides and identifying lines of enquiry specific to each study community.

Study population

The focus groups recruited young and heavy drinkers (assessed as above) in three contrasting study communities: an affluent urban community, and two deprived urban communities in Scotland. Focus group participants were recruited purposively (as described above), for the two population groups of interest, young binge drinkers aged 18-25 years and older heavy drinkers aged 30 years and over. At each wave 12 single-sex focus groups were

For peer review only - http://bmjopen.bmj.com/site/about/guidelines.xhtml

planned with an average of 5-6 participants in each group. The number and composition of the groups were selected to enable comparisons across two key study dimensions: types of drinker and socioeconomic status; and to facilitate open and free discussion allowing each participant the time to express their views.

One-to-one semi-structured interviews were conducted at each wave with key stakeholders from professional groups including representatives from licensing bodies, police, youth services, general practice and alcohol and drug treatment services.

Analysis plan

Focus group discussions and interviews were recorded with participants' permission and transcribed. Data will be coded thematically to allow the sharing of data across the research team and to enable identification of typical quotes, common reasoning and deviant or contradictory cases. Data from the interviews will be triangulated with the findings to emerge from the focus groups to provide a more vivid and rounded picture of community expectations, the implementation process and the legislation's short-term impact. Together these data will help to illuminate the findings to emerge from other study components.

Dissemination plan

Given the policy relevance of this work, dissemination will be targeted to academic, policy and public audiences using tailored outputs.

Academic papers will include:

1. EDs study: the impact of minimum unit pricing on alcohol-related attendances at EDs
2. SHCs study: changes in intoxicating substance use amongst young people attending SHCs following minimum unit pricing
3. Qualitative study: public expectations and understandings of minimum unit pricing, and changes in social experiences of alcohol use following its introduction

Further papers may be produced, dependent on the timing of data collection and following investigation for population subgroup-specific effects.

A series of short policy briefings and presentations will be produced to summarise both quantitative and qualitative findings.

These will be primarily aimed at policymakers within the Scottish and UK Governments, for example the Department of Health, Public Health England, Public Health Wales, the Public Health Agency in Northern Ireland, and NHS Health Scotland, but will be disseminated more broadly, subject to demand. The work will also be included in the review report NHS Health Scotland will produce for the Scottish Parliament and Ministers. This will provide evidence for Ministers' and the Scottish Parliament's decision about whether to renew the MUP legislation or allow it to fall under the 'sunset clause'. The academic and health and social care policy communities will be targeted through conference presentations.

Public audiences will be engaged through the production of plain-language summaries. These will be made available through a dedicated study website

For peer review only - http://bmjopen.bmj.com/site/about/guidelines.xhtml

MUP protocol paper v6 07.12.18

(http://sphsu.mrc.ac.uk/mup) which research participants will be signposted to (via the participant information leaflet). Posters summarising key results will be made available to study sites, so that findings of the research can be seen by both staff and patients. Broader public engagement will be achieved through an active mass media communication strategy, supported by ancillary use of social media when appropriate.

Data storage

Except as required for analysis, we have ensured that the electronic questionnaire data, consent forms, and data linkage forms are stored separately in three secure databases to maintain full confidentiality for these data.

Any paper-based data collected for the EDs study are stored, de-identified, in locked storage areas with strictly controlled access and handled according to MRC Good Research Practice Guidelines. Consents to participation in the study and to data linkage are stored separately in a dedicated secured area. Similarly, paper consent and data linkage forms are stored separately in a dedicated secured area.

SHCs data contain no personal identifiers, and are stored securely in a locked storage room and, when entered electronically, in a secure database.

Data Management

The original data were imported for data management and the original files backed up unaltered on a network drive only accessible to the project team. Data cleaning, manipulations and analysis were performed in separate coding/syntax files. We will provide well documented (i.e. with extensive commenting) syntax to ensure that all processing of data and results are reproducible.

Ethics review determined study integrity to be dependent upon retaining the anonymity of study participants and areas, including comparator sites. Necessary measures concern non-disclosure and protection of all local identifiers relating to local organisations (including health board areas, council areas and hospital sites) and named individuals and contact details of those associated with these and other local organisations. The protected period will apply from the start of the study to 1st September 2030.

Storage and use of data after the end of the study

Long term storage of data is at Iron Mountain, a commercial data storage facility. All data will be held in secure conditions with references for the contents of the sealed boxes held on a database for paper-held data. For electronic data secure electronic archives are used. Access to the data is controlled and permission from the data controller for the study is required to access long-term archived data. The electronic data will be archived at Glasgow University and destroyed by 1 September 2030 (ten years after the end of the study grant) as stated in R&D applications to research sites.

Strict data protection policies will be followed as outlined in the University of Glasgow's data protection policy.[77] The data will be worked from and stored upon a secure protected server (only accessible to the project team). Upon completion of the project, the data will then be

For peer review only

For peer review only - http://bmjopen.bmj.com/site/about/guidelines.xhtml

archived in line with University of Glasgow University guidance on data archiving and the MRC's 'Personal Information in Medical Research' guidance document.

Data Statement

Data will be held by the University of Glasgow Social and Public Health Sciences Unit.

Acknowledgements

PD is supported by South London and Maudsley (SLaM) NHS Foundation Trust and by the National Institute for Health Research (NIHR) Biomedical Research Centre (BRC) for Mental Health at King's College London and SLaM. CD is supported by the NIHR Collaborations for Leadership in Applied Health Research South London at King's College Hospital Foundation Trust, the NIHR BRC for Mental Health at King's College London and SLaM, and is in receipt of an NIHR Senior Investigator Award. The views expressed here are those of the authors and do not necessarily reflect the views of the Department of Health and Social Care or NIHR.

The iPad application (app) used for data collection in EDs was developed by Codeface Ltd.

The authors would like to thank the following NHS colleagues for their assistance with this work:

EDs

Dr Samantha Perry, Consultant in Emergency Medicine.

Dr Michele Open, Consultant in Emergency Medicine.

Dr Lynn Owens, Nurse Consultant.

Dr Christopher Yap, Consultant in Emergency Medicine.

SHCs

Dr Salmon Omokanye, Consultant in Genitourinary Medicine.

Dr Gabriel Schembri, Consultant in Genitourinary Medicine.

Dr Sarah Allstaff, Consultant Physician (GUM).

Dr Duncan McCormick, Consultant in Public Health Medicine.

Dr Pauline McGough, Clinical Director & Lead Clinician for Sexual Health.

Dr Janet Wilson, Consultant in Genitourinary Medicine.

For peer review only - http://bmjopen.bmj.com/site/about/guidelines.xhtml

In addition, we are particularly grateful to the Population Health Research Facility, and its predecessor, the Social and Public Health Sciences Surveys Unit at the University of Glasgow for their help with non-salary project costing, study training set up, study documents design, study operations set up, data entry, and data management.

Author Contributions

SVK wrote the first draft of the protocol. All authors made substantial contributions to the design of the research, critically revised the paper and approved the final manuscript.

Funding

This study was funded by the NIHR Public Health Research Programme (11/3005/40).

The Social and Public Health Sciences Unit is funded by the Medical Research Council (MC_UU_12017/13 & MC_UU_12017/15) and Scottish Government Chief Scientist Office (SPHSU13 & SPHSU15). SVK acknowledges funding from a NRS Senior Clinical Fellowship (SCAF/15/02).

Department of Health and Social Care disclaimer

The views expressed are those of the authors and not necessarily those of the NHS, the NIHR or the Department of Health and Social Care.

Competing interests

CB, GM and LG are members of the Scottish-Government funded Monitoring and Evaluating Scotland's Alcohol Strategy (MESAS) evaluation. The remaining authors declare that they have no conflicts of interest.

Ethics approval

Ethical approval has been obtained from the NHS through the Scotland A Research Ethics Committee for study components 1 and 2, REC references are 12/SS/0120 and 12/SS/0121.

The ethics approval for component 3 was obtained in 2012 from the University of Stirling Management School ethics Committee (Application numbers 32 and 33).

For peer review only

MUP protocol paper v6 07.12.18

References

1. Burgess L. Alcohol (Minimum Pricing) (Scotland) Bill: Passage of the Bill. Edinburgh, 2012.
2. Scottish Parliament. Alcohol (Minimum Pricing) (Scotland) Act 2012 (asp 4). Edinburgh: The Stationary Office Limited, 2012.
3. Katikireddi SV, Hilton S, Bonell C, et al. Understanding the Development of Minimum Unit Pricing of Alcohol in Scotland: A Qualitative Study of the Policy Process. *PLoS ONE* 2014;9(3):e91185. doi: 10.1371/journal.pone.0091185
4. Katikireddi SV, McLean JA. Introducing a minimum unit price for alcohol in Scotland: considerations under European Law and the implications for European public health. *The European Journal of Public Health* 2012;22(4):457-58. doi: 10.1093/eurpub/cks091
5. Katikireddi S V, Rice P, Holmes J, et al. Lessons from Scotland's experience of minimum unit pricing of alcohol. In: Giesbrecht N, Bosma L M, eds. Preventing Alcohol-Related Problems : Evidence and Community-Based Initiatives. Washington DC: American Public Health Association 2017.
6. Beeston C, Robinson M, Craig N, et al. Monitoring and Evaluating Scotland's Alcohol Strategy. Setting the Scene: Theory of change and baseline picture. Edinburgh: NHS health Scotland, 2011.
7. Abajobir AA, Abate KH, Abbafati C, et al. Global, regional, and national comparative risk assessment of 84 behavioural, environmental and occupational, and metabolic risks or clusters of risks, 1990-2016: a systematic analysis for the Global Burden of Disease Study 2016. *The Lancet* 2017;390(10100):1345-422. doi: 10.1016/S0140-6736(17)32366-8
8. Leon DA, McCambridge J. Liver cirrhosis mortality rates in Britain from 1950 to 2002: an analysis of routine data. *The Lancet* 2006;367(9504):52-56. doi: 10.1016/s0140-6736(06)67924-5
9. Tod E, Grant I, Wyper G, et al. Hospital admissions, deaths and overall burden of disease attributable to alcohol consumption in Scotland. Glasgow: Scottish Public Health Observatory/NHS Health Scotland, 2018.
10. Casswell S, Thamarangsi T. Reducing harm from alcohol: call to action. *The Lancet* 2009;373(9682):2247-57.
11. Beaglehole R, Bonita R. Alcohol: a global health priority. *The Lancet* 2009;373(9682):2173-74.
12. Gilmore I. Action needed to tackle a global drink problem. *The Lancet* 2009;373(9682):2174-76.
13. Room R, Babor T, Rehm J. Alcohol and public health. *The Lancet* 2005;365(9458):519-30.
14. World Health Organisation. Global status report on alcohol and health Luxembourg, 2014.
15. Holder HD. Alcohol and the Community: A Systems Approach to Prevention. Cambridge: Cambridge University Press 1998.
16. Babor T, Caetano R, Casswell S, et al. Alcohol: No ordinary commodity: Research and public policy. 2nd ed. Oxford: Oxford University Press 2010.
17. Herttua K, Mäkelä P, Martikainen P. Differential trends in alcohol-related mortality: a register-based follow-up study in Finland in 1987–2003. *Alcohol and Alcoholism* 2007;42(5):456-64. doi: 10.1093/alcalc/agl099
18. Mäkelä P, Valkonen T, Martelin T. Contribution of deaths related to alcohol use to socioeconomic variation in mortality: register based follow up study. *BMJ* 1997;315(7102):211-16. doi: 10.1136/bmj.315.7102.211
19. Leyland A, Dundas R, McLoone P, et al. Cause-specific inequalities in mortality in Scotland: two decades of change. A population-based study. *BMC Public Health* 2007;7(1):172.
20. Mackenbach JP, Kulhánová I, Bopp M, et al. Inequalities in Alcohol-Related Mortality in 17 European Countries: A Retrospective Analysis of Mortality Registers. *PLoS Med* 2015;12(12):e1001909. doi: 10.1371/journal.pmed.1001909
21. Katikireddi SV, Whitley E, Lewsey J, et al. Socioeconomic status as an effect modifier of alcohol consumption and harm: analysis of linked cohort data. *The Lancet Public Health* 2017;2(6):e267-e76.

For peer review only - http://bmjopen.bmj.com/site/about/guidelines.xhtml

22. Meier PS, Holmes J, Angus C, et al. Estimated Effects of Different Alcohol Taxation and Price Policies on Health Inequalities: A Mathematical Modelling Study. *PLoS Med* 2016;13(2):e1001963. doi: 10.1371/journal.pmed.1001963
23. Holmes J, Meng Y, Meier PS, et al. Effects of minimum unit pricing for alcohol on different income and socioeconomic groups: a modelling study. *The Lancet* 2014;383(9929):1655-64. doi: http://dx.doi.org/10.1016/S0140-6736(13)62417-4
24. Booth A, Meier P, Stockwell T, et al. Independent Review of the Effects of Alcohol Pricing and Promotion - Part A: Systematic Reviews. Sheffield: ScHARR, University of Sheffield, 2008.
25. Wagenaar AC, Salois MJ, Komro KA. Effects of beverage alcohol price and tax levels on drinking: a meta-analysis of 1003 estimates from 112 studies. *Addiction* 2009;104(2):179-90. doi: 10.1111/j.1360-0443.2008.02438.x
26. Huaung CD. Econometric models of alcohol demand in the UK. London: Government Economic Service, 2003.
27. Wagenaar AC, Tobler AL, Komro KA. Effects of Alcohol Tax and Price Policies on Morbidity and Mortality: A Systematic Review. *American Journal of Public Health* 2010;100(11):2270-78. doi: 10.2105/AJPH.2009.186007
28. Elder RW, Lawrence B, Ferguson A, et al. The Effectiveness of Tax Policy Interventions for Reducing Excessive Alcohol Consumption and Related Harms. *American Journal of Preventive Medicine* 2010;38(2):217-29. doi: 10.1016/j.amepre.2009.11.005
29. World Health Organization. Global strategy to reduce the harmful use of alcohol. Geneva, 2010.
30. Chisholm D, Moro D, Bertram M, et al. Are the “Best Buys” for Alcohol Control Still Valid? An Update on the Comparative Cost-Effectiveness of Alcohol Control Strategies at the Global Level. *Journal of Studies on Alcohol and Drugs* 2018;79(4):514-22. doi: 10.15288/jsad.2018.79.514
31. Burton R, Henn C, Lavoie D, et al. A rapid evidence review of the effectiveness and cost-effectiveness of alcohol control policies: an English perspective. *The Lancet* 2017;389(10078):1558-80. doi: https://doi.org/10.1016/S0140-6736(16)32420-5
32. Babor T, Caetano R, Casswell S, et al. Controlling affordability: Pricing and taxation. Alcohol: No ordinary commodity: Research and public policy. Oxford: Oxford University Press 2010:109-26.
33. UK Government. Tax on shopping and services 2018 [Available from: https://www.gov.uk/tax-on-shopping/alcohol-tobacco accessed 15/11/2018.
34. House of Commons Health Committee. Alcohol: First Report of Session 2009–10. London: House of Commons, 2009.
35. Health and Sport Committee. 2nd Report, 2012 (Session 4): Stage 1 Report on the Alcohol (Minimum Pricing) (Scotland) Bill. Edinburgh: Scottish Parliament, 2012.
36. Black H, Gill J, Chick J. The price of a drink: levels of consumption and price paid per unit of alcohol by Edinburgh's ill drinkers with a comparison to wider alcohol sales in Scotland. *Addiction* 2011;106(4):729-36. doi: 10.1111/j.1360-0443.2010.03225.x
37. Crawford MJ, Parry AMH, Weston ARW, et al. Relationship Between Price Paid for Off-Trade Alcohol, Alcohol Consumption and Income in England: A Cross-Sectional Survey. *Alcohol and Alcoholism* 2012;47(6):738-42. doi: 10.1093/alcalc/ags091
38. Purshouse RC, Meier PS, Brennan A, et al. Estimated effect of alcohol pricing policies on health and health economic outcomes in England: an epidemiological model. *The Lancet* 2010;375(9723):1355-64. doi: doi: 10.1016/S0140-6736(10)60058-X
39. Meng Y, Purshouse R, Brennan A, et al. Model-based Appraisal of Alcohol Minimum Pricing and Off-Licensed trade discount bans in Scotland using the Sheffield Alcohol Policy Model (v 2): An update based on newly available data. Sheffield: Scharr, University of Sheffield, 2010.
40. Angus C, Holmes J, Pryce R, et al. Model-based appraisal of the comparative impact of Minimum Unit Pricing and taxation policies in Scotland. Sheffield: University of Sheffield, 2016.

For peer review only - http://bmjopen.bmj.com/site/about/guidelines.xhtml

41. Bennetts R. IAS Briefing Paper: Use of Alcohol as a Loss-Leader. St Ives: Institute of Alcohol Studies, 2008.
42. Stockwell T, Leng J, Sturge J. Alcohol Pricing and Public Health in Canada: Issues and Opportunities. British Columbia: Centre for Addictions Research of BC, 2006.
43. Thompson K, Stockwell T, Wettlaufer A, et al. Minimum alcohol pricing policies in practice: A critical examination of implementation in Canada. *Journal of Public Health Policy* 2017;38(1):39-57. doi: 10.1057/s41271-016-0051-y
44. Katikireddi SV, Smith KE. Minimum unit pricing of alcohol in Scotland. In: Hill M, ed. Studying Public Policy. Bristol: Policy Press 2014.
45. Stockwell T, Zhao J, Martin G, et al. Minimum Alcohol Prices and Outlet Densities in British Columbia, Canada: Estimated Impacts on Alcohol-Attributable Hospital Admissions. *American Journal of Public Health* 2013:e1-e7. doi: 10.2105/ajph.2013.301289
46. Stockwell T, Zhao J, Giesbrecht N, et al. The raising of minimum alcohol prices in Saskatchewan, Canada: impacts on consumption and implications for public health. *Am J Public Health* 2012;102(12):e103-10. doi: 10.2105/AJPH.2012.301094 [doi] [published Online First: 2012/10/20]
47. Stockwell T, Auld MC, Zhao J, et al. Does minimum pricing reduce alcohol consumption? The experience of a Canadian province. *Addiction* 2012;107(5):912-20. doi: 10.1111/j.1360-0443.2011.03763.x
48. Stockwell T, Zhao J, Sherk A, et al. Assessing the impacts of Saskatchewan's minimum alcohol pricing regulations on alcohol-related crime. *Drug and Alcohol Review* 2016:n/a-n/a. doi: 10.1111/dar.12471
49. Zhao J, Stockwell T. The impacts of minimum alcohol pricing on alcohol attributable morbidity in regions of British Colombia, Canada with low, medium and high mean family income. *Addiction* 2017;112(11):1942-51. doi: doi:10.1111/add.13902
50. Stockwell T, Zhao J, Marzell M, et al. Relationships Between Minimum Alcohol Pricing and Crime During the Partial Privatization of a Canadian Government Alcohol Monopoly. *Journal of Studies on Alcohol and Drugs* 2015;76(4):628-34. doi: 10.15288/jsad.2015.76.628
51. Sherk A, Stockwell T, Callaghan RC. The effect on emergency department visits of raised alcohol minimum prices in Saskatchewan, Canada. *Drug and Alcohol Review* 2018;37(S1) doi: doi:10.1111/dar.12670 [published Online First: 12/02/2018]
52. McKee M. Alcohol in Russia. *Alcohol and Alcoholism* 1999;34(6):824-29. doi: 10.1093/alcalc/34.6.824
53. Holden C, Hawkins B. 'Whisky gloss': The alcohol industry, devolution and policy communities in Scotland. *Public Policy and Administration* 2012 doi: 10.1177/0952076712452290
54. Katikireddi SV, Bond L, Hilton S. Changing Policy Framing as a Deliberate Strategy for Public Health Advocacy: A Qualitative Policy Case Study of Minimum Unit Pricing of Alcohol. *Milbank Quarterly* 2014;92(2):250-83.
55. Hawkins B, Holden C. Framing the alcohol policy debate: industry actors and the regulation of the UK beverage alcohol market. *Critical Policy Studies* 2013;7(1):53-71. doi: 10.1080/19460171.2013.766023
56. McCambridge J, Hawkins B, Holden C. Industry Use of Evidence to Influence Alcohol Policy: A Case Study of Submissions to the 2008 Scottish Government Consultation. *PLoS Medicine* 2013;10(4):e1001431. doi: 10.1371/journal.pmed.1001431
57. Hawkins B, Holden C. 'Water dripping on stone'? Industry lobbying and UK alcohol policy. *Policy & Politics* 2014;42(1):55-70. doi: 10.1332/030557312x655468
58. Holden C, Hawkins B. 'Whisky gloss': The alcohol industry, devolution and policy communities in Scotland. *Public Policy and Administration* 2013;28(3):253-73. doi: 10.1177/0952076712452290

For peer review only - http://bmjopen.bmj.com/site/about/guidelines.xhtml

59. Katikireddi SV, Bond L, Hilton S. Perspectives on Econometric Modelling to Inform Policy: A UK Qualitative Case Study of Minimum Unit Pricing of Alcohol. *The European Journal of Public Health* 2014;24(3):490-95. doi: doi:10.1093/eurpub/ckt206
60. Katikireddi SV, Hilton S, Bond L. The role of the Sheffield model on the minimum unit pricing of alcohol debate: the importance of a rhetorical perspective. *Evidence and Policy: A Journal of Research, Debate and Practice* 2016;12(4):521-39.
61. Wood K, Patterson C, Katikireddi SV, et al. Harms to 'others' from alcohol consumption in the minimum unit pricing policy debate: a qualitative content analysis of UK newspapers (2005–12). *Addiction* 2014;109:578-84. doi: 10.1111/add.12427
62. Katikireddi SV, Hilton S. How did policy actors use mass media to influence the Scottish alcohol minimum unit pricing debate? Comparative analysis of newspapers, evidence submissions and interviews. *Drugs: Education, Prevention, and Policy* 2014;22(2):125-34. doi: 10.3109/09687637.2014.977228
63. Patterson C, Katikireddi SV, Wood K, et al. Representations of minimum unit pricing for alcohol in UK newspapers: a case study of a public health policy debate. *Journal of Public Health* 2015;37(1):40-49.
64. Hilton S, Wood K, Patterson C, et al. Implications for alcohol minimum unit pricing advocacy: What can we learn for public health from UK newsprint coverage of key claim-makers in the policy debate? *Social Science & Medicine* 2014;102(0):157-64. doi: http://dx.doi.org/10.1016/j.socscimed.2013.11.041
65. Lonsdale A, Hardcastle S, Hagger M. A minimum price per unit of alcohol: A focus group study to investigate public opinion concerning UK government proposals to introduce new price controls to curb alcohol consumption. *BMC Public Health* 2012;12(1):1023.
66. Pechey R, Burge P, Mentzakis E, et al. Public acceptability of population-level interventions to reduce alcohol consumption: A discrete choice experiment. *Social Science and Medicine* 2014;113(0):104-09. doi: http://dx.doi.org/10.1016/j.socscimed.2014.05.010
67. Li J, Lovatt M, Eadie D, et al. Public attitudes towards alcohol control policies in Scotland and England: Results from a mixed-methods study. *Social Science & Medicine* 2017;177(Supplement C):177-89. doi: https://doi.org/10.1016/j.socscimed.2017.01.037
68. O'May F, Gill J, Black H, et al. Heavy Drinkers' Perspectives on Minimum Unit Pricing for Alcohol in Scotland:A Qualitative Interview Study. *SAGE Open* 2016;6(3):2158244016657141. doi: 10.1177/2158244016657141
69. Craig P, Katikireddi SV, Leyland AH, et al. Natural experiments: An overview of methods, approaches and contribution to public health intervention research. *Annual Review of Public Health* 2017;38(1):39-56.
70. Craig P, Cooper C, Gunnell D, et al. Using natural experiments to evaluate population health interventions: new Medical Research Council guidance. *JECH* 2012;66:1182-6. doi: 10.1136/jech-2011-200375
71. NHS Health Scotland. Monitoring and Evaluating Scotland's Alcohol Strategy (MESAS): Briefing paper. December 2009 Glasgow, 2009.
72. Bonell C, Jamal F, Melendez-Torres GJ, et al. 'Dark logic': theorising the harmful consequences of public health interventions. *Journal of Epidemiology and Community Health* 2014 doi: 10.1136/jech-2014-204671
73. Hodgson R, Alwyn T, John B, et al. THE FAST ALCOHOL SCREENING TEST. *Alcohol and Alcoholism* 2002;37(1):61-66. doi: 10.1093/alcalc/37.1.61
74. Drummond DC, Phillips, T., Coulton, S., Barnaby, B., Keating, S., Sabri, R. & Moloney, J. Saturday Night and Sunday Morning: the 2003 twenty-four hour national survey of alcohol-related attendances at accident and emergency departments in England. Final report to the Cabinet Office. London: Institute of Psychiatry, 2003.

For peer review only - http://bmjopen.bmj.com/site/about/guidelines.xhtml

75. NHS Quality Improvement Scotland. NHS Quality Improvement Scotland. Scottish Emergency Department Alcohol Audit: Understanding Alcohol Misuse in Scotland – Final Report. Edinburgh, 2008.
76. Drummond D, Deluca P, Coulton S, et al. The Effectiveness of Alcohol Screening and Brief Intervention in Emergency Departments: A Multicentre Pragmatic Cluster Randomized Controlled Trial. . *PLoS ONE* 2014;9(6):e99463. doi: https://doi.org/10.1371/journal.pone.0099463
77. University of Glasgow, Data Protection Policy 2006 [Available from: http://www.gla.ac.uk/services/dpfoioffice/policiesandprocedures/dpa-policy/.

For peer review only

STROBE 2007 (v4) Statement—Checklist of items that should be included in reports of *cross-sectional studies*

Section/Topic	Item #	Recommendation	Reported on page #
**Title and abstract**	1	(*a*) Indicate the study's design with a commonly used term in the title or the abstract	Title p1
		(*b*) Provide in the abstract an informative and balanced summary of what was done and what was found	Abstract p2
**Introduction**			
Background/rationale	2	Explain the scientific background and rationale for the investigation being reported	Pp4-6
Objectives	3	State specific objectives, including any prespecified hypotheses	EDs Pp7-8,<br>SHCs p11,<br>Qualitative pp12-13
**Methods**			
Study design	4	Present key elements of study design early in the paper	EDs/SHCs P5,<br>Qualitative p13
Setting	5	Describe the setting, locations, and relevant dates, including periods of recruitment, exposure, follow-up, and data collection	EDs Pp7-9,<br>SHCs p11-12,<br>Qualitative p13
Participants	6	(*a*) Give the eligibility criteria, and the sources and methods of selection of participants	EDs P8,<br>SHCs p11,<br>Qualitative P13
Variables	7	Clearly define all outcomes, exposures, predictors, potential confounders, and effect modifiers. Give diagnostic criteria, if applicable	EDs Pp9-10,<br>SHCs p14,
Data sources/ measurement	8*	For each variable of interest, give sources of data and details of methods of assessment (measurement). Describe comparability of assessment methods if there is more than one group	EDs p9<br>SHCs pp11-12<br>Qualitative pp13-14
Bias	9	Describe any efforts to address potential sources of bias	EDs pp9-10<br>SHCs p12<br>Qualitative p14
Study size	10	Explain how the study size was arrived at	EDs p10<br>SHCs p12

For peer review only

			Qualitative p13
Quantitative variables	11	Explain how quantitative variables were handled in the analyses. If applicable, describe which groupings were chosen and why	EDs p10<br>SHCs p12<br>Qualitative n/a
Statistical methods	12	(*a*) Describe all statistical methods, including those used to control for confounding	EDs p10<br>SHCs p12<br>Qualitative n/a
		(*b*) Describe any methods used to examine subgroups and interactions	EDs p10<br>SHCs p12<br>Qualitative n/a
		(*c*) Explain how missing data were addressed ***NOT applicable as this is a protocol paper***	EDs<br>SHCs<br>Qualitative
		(*d*) If applicable, describe analytical methods taking account of sampling strategy ***NOT applicable from here on as this is a protocol paper***	EDs<br>SHCs<br>Qualitative
		(*e*) Describe any sensitivity analyses ***NOT applicable from here on as this is a protocol paper***	EDs<br>SHCs<br>Qualitative
**Results**		***NOT applicable from here on as this is a protocol paper***	
Participants	13*	(a) Report numbers of individuals at each stage of study—eg numbers potentially eligible, examined for eligibility, confirmed eligible, included in the study, completing follow-up, and analysed	EDs<br>SHCs<br>Qualitative
		(b) Give reasons for non-participation at each stage	EDs<br>SHCs<br>Qualitative
		(c) Consider use of a flow diagram	EDs<br>SHCs<br>Qualitative
Descriptive data	14*	(a) Give characteristics of study participants (eg demographic, clinical, social) and information on exposures and potential confounders	EDs<br>SHCs

For peer review only

			Qualitative
		(b) Indicate number of participants with missing data for each variable of interest	EDs<br>SHCs<br>Qualitative
Outcome data	15*	Report numbers of outcome events or summary measures	EDs<br>SHCs<br>Qualitative
Main results	16	(*a*) Give unadjusted estimates and, if applicable, confounder-adjusted estimates and their precision (eg, 95% confidence interval). Make clear which confounders were adjusted for and why they were included	EDs<br>SHCs<br>Qualitative
		(*b*) Report category boundaries when continuous variables were categorized	EDs<br>SHCs<br>Qualitative
		(*c*) If relevant, consider translating estimates of relative risk into absolute risk for a meaningful time period	EDs<br>SHCs<br>Qualitative
Other analyses	17	Report other analyses done—eg analyses of subgroups and interactions, and sensitivity analyses	EDs<br>SHCs<br>Qualitative
**Discussion**			
Key results	18	Summarise key results with reference to study objectives	EDs<br>SHCs<br>Qualitative
Limitations	19	Discuss limitations of the study, taking into account sources of potential bias or imprecision. Discuss both direction and magnitude of any potential bias	EDs<br>SHCs<br>Qualitative
Interpretation	20	Give a cautious overall interpretation of results considering objectives, limitations, multiplicity of analyses, results from similar studies, and other relevant evidence	EDs<br>SHCs<br>Qualitative
Generalisability	21	Discuss the generalisability (external validity) of the study results	EDs<br>SHCs

For peer review only

			Qualitative
**Other information**			
Funding	22	Give the source of funding and the role of the funders for the present study and, if applicable, for the original study on which the present article is based	EDs/SHCs/Qualitative P17

*Give information separately for cases and controls in case-control studies and, if applicable, for exposed and unexposed groups in cohort and cross-sectional studies.

Note: An Explanation and Elaboration article discusses each checklist item and gives methodological background and published examples of transparent reporting. The STROBE checklist is best used in conjunction with this article (freely available on the Web sites of PLoS Medicine at http://www.plosmedicine.org/, Annals of Internal Medicine at http://www.annals.org/, and Epidemiology at http://www.epidem.com/). Information on the STROBE Initiative is available at www.strobe-statement.org.

For peer review only

BMJ Open

Evaluating possible intended and unintended consequences of the implementation of alcohol minimum unit pricing (MUP) in Scotland: a natural experiment protocol

Journal:	*BMJ Open*
Manuscript ID	bmjopen-2018-028482.R1
Article Type:	Protocol
Date Submitted by the Author:	27-Mar-2019
Complete List of Authors:	Katikireddi, Srinivasa; University of Glasgow School of Life Sciences, MRC/CSO Social & Public Health Sciences Unit<br>Beeston, Clare; NHS Health Scotland,<br>Millard, Andrew; University of Glasgow, MRC/CSO Social and Public Health Sciences Unit<br>Forsyth, Ross; University of Glasgow School of Life Sciences, MRC/CSO Social & Public Health Sciences Unit<br>Deluca, Paolo; King's College London, Addictions<br>Drummond, Colin; Kings College London, Institute of Psychiatry<br>Eadie, Douglas; Institute for Social Marketing, School of Health Sciences<br>Graham, Lesley; ISD Scotland<br>Hilton , Shona; University of Glasgow, MRC/CSO Social & Public Health Sciences Unit<br>Ludbrook, Anne; University of Aberdeen, Health Economics Research Unit<br>McCartney, Gerry; NHS Health Scotland<br>Phillips, Thomas; University of Hull, Faculty of Health Sciences Institute for Clinical and Applied Health Research (ICAHR)<br>Stead, Martine; University of Stirling and the Open University, Institute for Social Marketing<br>Ford, Allison; University of Stirling, Institute for Social Marketing<br>Bond, Lyndal; Australian Health Policy Collaboration, Victoria University,<br>Leyland, Alastair; MRC/CSO Social and Public Health Sciences Unit, University of Glasgow,
**Primary Subject Heading**:	Addiction
Secondary Subject Heading:	Public health
Keywords:	ACCIDENT & EMERGENCY MEDICINE, PUBLIC HEALTH, SEXUAL MEDICINE

SCHOLARONE™
Manuscripts

Evaluating possible intended and unintended consequences of the implementation of alcohol minimum unit pricing (MUP) in Scotland: a natural experiment protocol

Srinivasa Vittal Katikireddi: Vittal.Katikireddi@glasgow.ac.uk [1, 2]

Clare Beeston: clare.beeston@nhs.net [3]

Andrew Denis Millard: Andrew.millard@glasgow.ac.uk [1]

Ross Forsyth: Ross.Forsyth@glasgow.ac.uk [1]

Paolo Deluca: paolo.deluca@kcl.ac.uk [4]

Colin Drummond: colin.drummond@kcl.ac.uk [4]

Douglas Eadie: douglas.eadie@stir.ac.uk [5]

Lesley Graham: lesley.graham@nhs.net [6]

Shona Hilton: Shona.Hilton@glasgow.ac.uk [1]

Anne Ludbrook: a.ludbrook@abdn.ac.uk [7]

Gerry McCartney: gmccartney@nhs.net [3]

Thomas Phillips: Thomas.Phillips@hull.ac.uk [4, 8]

Martine Stead: martine.stead@stir.ac.uk [5]

Allison Ford: a.j.ford@stir.ac.uk [5]

Lyndal Bond: Lyndal.Bond@vu.edu.au [9]

Alastair H Leyland: Alastair.Leyland@glasgow.ac.uk [1]

[1] MRC/CSO Social and Public Health Sciences Unit, University of Glasgow

[2] Department of Public Health and Health Policy, NHS Lothian

[3] NHS Health Scotland

[4] National Addiction Centre, Institute of Psychiatry, Psychology and Neuroscience, King's College London

[5] Institute for Social Marketing, University of Stirling

[6] Information Services Division, NHS National Services Scotland

[7] Health Economics Research Unit, University of Aberdeen

[8] Institute for Clinical and Applied Health Research (ICAHR), University of Hull

[9] Australian Health Policy Collaboration, Victoria University, Australia

Correspondence to

Dr Andrew D. Millard: Andrew.millard@glasgow.ac.uk

For peer review only - http://bmjopen.bmj.com/site/about/guidelines.xhtml

MUP protocol paper v3 27.03.19

Keywords: alcohol, pricing, policy, evaluation

Abstract

Introduction

Scotland is the first country to carry out a national implementation of Minimum Unit Pricing (MUP) for alcohol. MUP aims to reduce alcohol-related harms, which are high in Scotland compared to Western Europe, and to improve health equalities. MUP is a minimum retail price per unit of alcohol. That approach targets high-risk alcohol users. This work is key to a wider evaluation that will determine whether MUP continues. There are three study components.

Methods and Analysis

Component 1 sampled an estimated 2800 interviewees at a baseline and each of two follow ups from four Emergency Departments in Scotland and Northern England. Research nurses administered a standardised survey to assess alcohol consumption and the proportion of attendances that were alcohol-related.

Component 2 covered six Sexual Health Clinics with similar timings and country allocation. A self-completion survey gathered information on potential unintended effects of MUP on alcohol source and drug use.

Using a natural experiment design and repeated cross-sectional audit, difference in difference between Scotland (intervention) and North England (control) will be tested for outcomes using regression adjusting for differences at baseline. Differential impacts by age, gender and socioeconomic position will be investigated.

Component 3 used focus groups with young people and heavy drinkers and interviews with stakeholders before and after MUP implementation. The focus groups will allow exploration of attitudes, experiences and behaviours, and the potential mechanisms by which impacts arise. The interviews will help characterise the implementation process.

Ethics and Dissemination

Study components 1 and 2 have been ethically approved by the NHS, and component 3 by the University of Stirling. Dissemination plans include peer-reviewed journal articles, presentations, policy maker briefings and, in view of high public interest and the high political profile of this flagship policy, communication with the public via media engagement and plain language summaries.

300/300 words

Main paper 5765 words

For peer review only - http://bmjopen.bmj.com/site/about/guidelines.xhtml

Strengths and limitations of this study

- This evaluation adopts multiple methods to help establish whether MUP has caused changes in alcohol-related attendances using a natural experiment design, which is the most appropriate for this topic as a randomized controlled trial would not be feasible.
- Our study exploits divergences in Scottish and English alcohol policy to evaluate the effectiveness of an all-beverage MUP for the first time, and evaluates both positive and possible negative impacts of minimum unit pricing of alcohol; such negative impacts might include use of other sources of alcohol, other substances, or reduction in money available for essentials.
- The Northern English control group is likely to be comparable to Scotland because of geographical proximity and similar levels of deprivation, but we also assess the external validity of our sample by reference to routine data on attendances at Emergency Departments and Sexual Health Clinics.
- There is the potential to follow up individuals through longer term data linkage, for which we will obtain respondents' permission, thus adding a cohort dimension to the initial cross-sectional approach.
- The main limitation is that the non-randomised design risks selection bias, for example in the differential selection of intoxicated attendees for interview.

For peer review only

Introduction

Minimum unit pricing (MUP) of alcohol is a novel public health policy that aims to reduce alcohol-related harms across the population. In May 2012, Scotland became the first country to pass legislation to introduce MUP without reference to beverage type, a politically high profile measure,[1-3] although some Canadian provinces have introduced beverage-specific MUP, as further described below. This paper describes the protocol for three associated studies that collect primary data to evaluate the impact of the policy in Scotland. After delays following legal challenges,[4 5] MUP legislation was implemented in May 2018 but it is due to cease in six years unless shown to be effective, under a 'sunset clause'. These studies are a key part of the evaluation planned by NHS Health Scotland, which will report to Scottish Ministers and the Scottish Parliament. The evaluation includes analyses by others of routinely collected administrative data.[6]

The overall aim of the study is to investigate the impacts of MUP on acute and chronic health harms (including by deprivation and gender and age subgroups). The study will also determine the extent to which specific unintended consequences occur. The evidence will be a key input to the decision whether the policy will continue.

Alcohol consumption is a major determinant of population health globally,[7] and the level of alcohol-related harm in Scotland is high compared to the rest of Western Europe.[8] Alcohol is associated with over 200 medical conditions including an increased risk of liver disease, heart disease, unintended pregnancy, sexually transmitted infections, some cancers and some injuries.[9-14] Its impact extends beyond the individual, with adverse effects on families, communities and the wider economy.[15 16] Furthermore, alcohol is known to be a major contributor to socioeconomic inequalities in health in the UK and elsewhere.[17-20] From a public health perspective, alcohol therefore has considerable potential as a modifiable risk factor to be addressed to help reduce health inequalities.[21-23]

A consistent body of international evidence demonstrates a negative relationship between price, consumption and alcohol-related harms.[24-28] The World Health Organization has identified changing the price of alcohol, and controls on promotion and availability, as key methods for addressing alcohol-related health harms.[29 30 31] Across much of the world, the best established mechanism for influencing price has been taxation through alcohol duty, although in many countries, including the UK, the primary purpose is revenue-raising for central government rather than achieving public health goals.[32] As a result of this secondary consideration of public health, alcohol duty may be poorly designed to address alcohol-related harms since duty typically varies by product in a manner not consistent with the associated harms arising from the product's consumption. At the time of writing tax on a litre of 7.5% ABV cider is 40 pence, on a litre of wine of the same strength tax would be 289 pence.[33]

A potentially complementary approach to increasing alcohol duty is the introduction of a floor price, such as MUP, below which alcohol should not be retailed.[34 35] MUP sets a minimum price per UK unit (eight grams) of pure alcohol. Epidemiological studies have found that drinkers at the greatest risk of harm tend to consume the cheapest (per unit) alcohol, thereby providing evidence that MUP may better target harmful drinkers in comparison to alcohol duty.[36 37] Econometric modelling studies suggest that MUP will result in a greater reduction in health harms compared to an equivalent rise in taxation under the UK's current system of

For peer review only - http://bmjopen.bmj.com/site/about/guidelines.xhtml

calculating alcohol duty.[21 22 38-40] In addition, setting a floor price minimises the potential for 'trading down' to cheaper drinks, given that alcoholic drinks below the floor price would no longer be legally available. This is particularly relevant since previously retailers have opted not to pass on alcohol duty increases to consumers, as indicated by the existence of below-cost products.[41]

Comparable interventions to MUP have been introduced elsewhere. The best known example is in Canada, where some Canadian provinces introduced a minimum price for selling specific beverages (also referred to as 'reference pricing') within the context of government-operated monopolies.[42 43] While some authors refer to these interventions as 'minimum pricing', the nature of this policy differs in some aspects from 'minimum unit pricing' as planned in Scotland.[3 5 44] In contrast to MUP which applies a uniform minimum price per unit across all beverage types, reference pricing imposes differing minimum prices that are determined by both alcohol strength and drink type. In addition, MUP in Scotland was introduced into a competitive alcohol market at a national level, in contrast to the locally applied government-owned monopolies in which reference pricing has been introduced. Important benefits of reference pricing in Canada have been demonstrated, with reductions in alcohol consumption, alcohol-attributable hospital admissions and crime observed.[45-50] However, a study focusing on Emergency Department (ED) visits found no reduction in overall visits for alcohol-related injuries, although alcohol-related motor vehicle injuries did fall.[51] Other broadly comparable pricing interventions, such as the abolition of cheap vodka within communist Russia, have similarly been associated with reductions in alcohol-related mortality.[52]

In Scotland, qualitative research has investigated the policy process through which MUP developed, including assessing the role of commercial interests, and seeking to identify transferable lessons for public health advocacy.[3 5 44 53-57] The influence of econometric modelling has been specifically investigated.[58 59]The dominant media discourses and the roles of different policy stakeholders in articulating arguments to the public have been explored using content analysis of newspaper reporting and trends in newspaper coverage have been tracked over time.[60-63] The views of the public and heavy drinkers around MUP have also been investigated.[64-67] There remained a need to investigate the views of policy stakeholders, the public, and heavy drinkers about MUP as implemented.

Empirical research gathering primary data specifically focused on MUP in Scotland remains limited. The introduction of MUP provides a unique opportunity to use a natural experiment to evaluate public health policy, and provide new real-world evidence on the effectiveness of MUP, where most evidence to date has been from modelling studies.[68 69] A broad programme of research is being co-ordinated by NHS Health Scotland, which will report to Scottish Ministers and the Scottish Parliament between five and six years after the start of the policy. This programme includes analyses of administrative data and alcohol sales data.[70] The protocol described here complements the NHS-led work and has been funded by the National Institute for Health Research (NIHR) Public Health Research programme to collect primary data in three associated studies.

Much of the burden of alcohol-related harm, especially amongst young people, arises from acute harm following high episodic consumption ('binge drinking'). These harms are of particular interest to policymakers as they may be associated with social disorder and violence. That in turn provides one justification for MUP, although the case for MUP

For peer review only - http://bmjopen.bmj.com/site/about/guidelines.xhtml

legislation in Scotland was fought (and won) on public health benefits. Comprehensive diagnostic data on ED alcohol-related attendances that do not result in admission are currently not adequately captured in administrative health data within the UK, so require primary research.

Our work focuses on both intended and possible unintended consequences of the intervention.[71] A number of potential risks arising from the introduction of MUP have been identified by policymakers, the alcohol industry and public health professionals.[54]

1. Consumers may switch to alternative sources of alcohol not subject to MUP so that the price paid does not increase. Such sources include both legal sources: internet sales from outside Scotland, legitimate cross-border purchase for own use, and home fermentation and illegal sources (counterfeit, and unlicensed sale of smuggled or stolen alcohol).
2. Increased alcohol-related harm could occur through substitution (e.g. to illicitly produced or industrial alcohol associated with greater toxicity) or changed drinking patterns (e.g. moving from regular drinking to binge drinking).
3. Displacement effects with reductions in alcohol-related harms potentially accompanied by increases in harms related to other substance use.
4. MUP could unfairly penalise deprived populations less able to absorb the additional financial cost and this may adversely affect access to essentials such as food and fuel.
5. MUP may have unintended effects on harmful drinkers who cannot reduce their consumption.
6. There may be adverse economic impacts on the Scottish alcohol industry retailers and/or manufacturers.

The ED and Sexual Health Clinics (SHC) studies described in this protocol will address the first four of these potential risks, although analysis of existing datasets by NHS Health Scotland will further address the potential impact on access to essential goods. A study has been commissioned from the University of Sheffield by NHS Health Scotland to assess the effects on harmful drinkers. The possible adverse economic impact on the Scottish alcohol industry will not be addressed by our project but will be monitored by the Scottish Government through the Monitoring and Evaluating Scotland's Alcohol Strategy (MESAS) studies managed by NHS Health Scotland.

It was not possible to collect sufficiently valid consumption data in the ED setting to assess alcohol consumption levels based on consumption owing to interview time constraints. Instead, we defined hazardous, harmful and dependent alcohol consumption using scores from the Fast Alcohol Screening Test (FAST).[72] The use of FAST to identify alcohol misuse (i.e. hazardous, harmful and dependent alcohol consumption) has been supported by national guidelines for the prevention of harmful drinking.[73] Derived from the AUDIT[74] the four item FAST was specifically developed and validated within ED as it is quick to administer and less disruptive in the busy ED setting.[72,75] The FAST has been found to be the most sensitive and accurate short alcohol screening tool commonly administered to identify alcohol misuse in adults attending ED.[76,77] and has been used in a number of clinical studies.[78,79] A score of ≥3 on the FAST indicates hazardous drinking. We are undertaking a parallel examination of The FAST screening tool to further discriminate alcohol use disorders in ED attendees. In our study MUP is theorised to reduce alcohol misuse (hazardous, harmful and dependent alcohol consumption), so reducing both acute and chronic alcohol-related harm, and therefore the numbers of alcohol-related attendances at both EDs and at

For peer review only - http://bmjopen.bmj.com/site/about/guidelines.xhtml

SHCs. Hazardous drinkers are expected to be more likely than harmful or dependent drinkers to present with acute harms (such as injuries and assaults), while harmful/dependent drinkers are likely to present with both acute conditions and acute consequences of chronic conditions (such as pancreatitis and gastritis). Therefore there may be an early impact on ED attendances relating to chronic as well as acute alcohol-related conditions.

Research questions

The more specific research questions and objectives are summarised in Table 1, with the contribution of each of the three study components stated.

Table1 Research Aims and Study Components

**Research aims (RAs)**	**Study components (C)**
RA1: To determine the impact of MUP on alcohol-related harms and drinking patterns for the overall population and by subgroups of interest (age, sex and socioeconomic position).	ED study of alcohol-related attendances (C1);<br><br>Survey of alcohol-related behaviours (consumption patterns, alcohol spend, source of alcohol, move to other substances) in SHCs (C2)
RA2: To determine the impact of MUP on non-alcohol substance use, and other unintended impacts, for the overall population and by subgroups of interest (age, sex and socioeconomic position).	Survey of alcohol-related behaviours in SHCs (C2);<br><br>Qualitative focus group study and stakeholders (C3)
RA3: To describe changes in experiences and norms towards MUP and alcohol use following the introduction of MUP by subgroups of interest (age, sex and socioeconomic position).	Qualitative focus groups with young people/heavy drinkers and interviews with stakeholders from public services (C3)

Another separately funded study is focusing on the impacts on people with alcohol dependence, including high rates of homelessness specifically. We therefore do not target this group for our qualitative work.

At the time of revising this paper for referees' comments, all data have been collected for all three components and analysis will shortly commence.

Methods and analysis

Component 1: Audit of alcohol-related attendances at EDs

Study design

For peer review only - http://bmjopen.bmj.com/site/about/guidelines.xhtml

This component is designed to quantify changes in alcohol-related attendances at ED settings as a result of MUP. The FAST is used to identify alcohol misuse in the ED. The component focuses on answering RA1, namely:

1. What are the impacts of MUP for alcohol on alcohol-related harms and drinking patterns (using the FAST) for ED attendees and by subgroups of interest (age, sex and deprivation)?
2. Does the effect of MUP vary dependent on the type of alcohol-related harm:
    a. Acute alcohol-related harms vs chronic alcohol-related harms?
    b. Broad diagnostic groups (based on coding systems used in EDs?
3. Does MUP affect alcohol consumption (on the basis of the FAST[72] score and alcohol misuse over the reporting period amongst people attending EDs (FAST $\geq$3)?
4. Does the MUP intervention effect size (assessed on a variety of measures including FAST) vary at the second and third time points?

Lower level outcomes are set out in the section on data collection below. To answer research aim 1, a natural experiment design employing a repeated cross-sectional audit of all alcohol-related attendances at EDs in two Scottish hospitals and two North of England comparator hospitals at three time points was most appropriate to assess causality. A Randomised Controlled Trial (RCT) was not feasible. The study methods and tools were based on previous studies used to quantify the national prevalence of alcohol-related attendances in EDs in England[79] and informed by the experience of the Scottish ED Alcohol Audit, carried out across 15-20 hospitals throughout mainland Scotland between October 2005 and June 2007.[80]

Study population

People aged 16 years and over attending EDs during data collection periods were approached. The study monitored, but excluded from interview:

- those acutely physically or mentally ill such that informed consent or full participation was impossible to obtain (including gross intoxication),
- those with whom it was impossible to adequately communicate (e.g. patients unable to speak English despite the use of an interpreter where available), and
- those who left the department before an approach was made or who were deemed by the research nurses as too threatening to approach.

Reasons for not approaching were recorded to address potential selection bias.

Data collection

Baseline data collection took place in February 2018 prior to the implementation of MUP on 1st May 2018. The second and third waves of data collection took place approximately eight and 12 months post baseline, and five and nine months post implementation, with care taken to avoid the inclusion of specific events that are expected to be associated with increased or decreased alcohol consumption, for example local holidays were checked to avoid

For peer review only - http://bmjopen.bmj.com/site/about/guidelines.xhtml

differences between groups. Each wave collected data over three weeks, and to maximise collection of both alcohol-related attendances and alcohol misuse, data collection occurred from 20.00 to 03.30 on Thursday to Sunday (i.e. 30 hours per week during this time period) and 09.00 to 16.30 on Monday to Wednesday (i.e. 22.5 hours per week during this time period).

During study data collection periods, patients were approached by a research nurse to seek permission to complete a short face-to-face interview. All eligible patients were approached, informed consent obtained, and a face-to-face interview based on a previously used patient questionnaire was administered electronically using a tablet (iPad).[78] An ED attendance database was maintained for all attendances during data collection periods to allow monitoring of patient recruitment.

The interview collected details on the following:

- basic demographic information (age; gender; ethnicity; marital, employment and housing status (including unstable housing status such as no fixed abode and hostel accommodation); postcode sector);
- attendance details (date and time, triage category, time and date of incident),
- how much alcohol was consumed in the past 24 hours;
- date, time and place of last drink;
- the largest number of drinks on any one day in the previous week;
- self-reported reason for attendance, such as assault, collapse etc;
- alcohol misuse in the past year (as assessed by the FAST[72]) and
- whether alcohol consumption changed over the previous 12 months.

In addition we asked whether interviewees thought their current ED attendance was related to their or someone else's drinking, and how many times they had attended any ED in the previous 12 months.

A note was made of the hospital unique identifier for the attendance if participants agreed to link their research interview data to their ED data. Research staff retrieved additional data from patient records on full postcode, triage category, discharge status and diagnoses at discharge (coded by ICD-10 or other ED coding systems in use locally). The consent procedure asked participants for separate permissions both for this and for data linkage to facilitate longitudinal follow-up. Additionally, aggregated data on age group, sex and diagnosis were sought for all attendees in the study time periods in order to allow an assessment of the representativeness of the interviewee sample.

Socioeconomic position and inequalities

Analysis by deprivation, age and sex will be undertaken to determine if the impact of MUP on alcohol-related attendances at EDs is differentially patterned. Assessment of alcohol-related attendances within the ED setting provides a broad range of deprivation, age, sex and drinking status categories. It therefore not only provides information on an important primary health outcome but also allows adequate investigation of the intervention's impact on important subgroups.

Outcome measures

For peer review only - http://bmjopen.bmj.com/site/about/guidelines.xhtml

The primary outcome will be the proportion of attendances that are alcohol-related, using a composite measure which defined the attendance as alcohol-related if any one (or more) of the following was true:

- patient self-reports attendance is alcohol-related;
- patient reports alcohol consumption in past 24 hours ≥8 units in men, ≥6 units in women; or
- patient not approached or interview terminated because too intoxicated with alcohol.

Secondary outcomes will be:

- the absolute number and proportion of alcohol-related attendances;
- alcohol misuse defined by the FAST score ≥3;
- mean FAST score by age, gender, and deprivation;
- prevalence of binge drinking in the past week, and
- self-reported reason for attendance.

Outcomes will be compared between intervention and control areas, adjusted for baseline attendances.

Power calculation

Based on the experience of the 24 hour audit of EDs in England,[79] and assuming that we would be able to recruit 50% of eligible ED attendees, we anticipated that the four sites (two Scotland, two England) would result in 940 recruits per week. Recruiting over 3 weeks pre- and post-implementation – giving a total sample size of 5,640 for the baseline and any one subsequent wave - would mean that we would be highly powered (>80%) to detect an effect size of ±5% in the proportion of alcohol-related attendances from an estimated 30% with 95% significance. We have used a base rate of 30% informed by the 24 hour audit of EDs in England[79] and assumed a 5% decrease would be of public health importance and may be expected based on current evidence.[79] For sub-group analyses, we would have good power (>80%) to detect an effect size of 0.23 on the FAST score among those from the most deprived quintile (estimated to be 25% of attendances) and an effect size of 0.27 among those aged 18-24 years (estimated to be 15% of attendances).

Statistical analysis

We will test for differences in the outcomes between the intervention and control groups using a regression model with ED included as a fixed effect, and with individuals nested within the ED of their attendance, before and after adjustment for relevant covariates including baseline levels of alcohol-related attendance, age, gender, deprivation quintile, and disease diagnostic categories. We will also attempt to determine the nature of the effect more precisely in terms of whether there is a dose-response effect according to the time since MUP was implemented (through a test of the significance of an interaction between time and intervention). We will test for interactions of the intervention with defined important covariates (including baseline levels of alcohol-related attendance, age, gender, deprivation quintile, and disease diagnostic categories) to investigate the possibility of differential intervention effects and will subsequently stratify the analyses if indicated.

For peer review only

Ethical arrangements

Trained nurse researchers approached and interviewed participants in order to ensure informed consent was obtained from study participants. The research team has considerable expertise with appropriately considering ethical issues in studies with similar methodology previously. [79,80] We followed standard procedures for obtaining informed consent. Participants received information on local alcohol services and national advice telephone lines in the participant information leaflet.

Component 2: Survey of alcohol-related behaviours in sexual health services

Young adults and high-risk alcohol users are under-represented in national surveys. SHCs were selected as the setting for data collection to provide adequate inclusion of deprived groups and young people. Understanding how MUP might change alcohol misuse patterns in this group and/or increase use of other substances is considered a priority by policymakers. SHCs provide a convenience-sampling frame to monitor changes in behaviour within a high-risk population that includes adequate representation of both deprivation and young age. This component contributes to RA1 and RA2, specifically to investigate, amongst a population at high-risk of alcohol and drug-related problems:

1. changes in alcohol misuse
2. how sources of alcohol consumption have changed following MUP
3. whether MUP has impacted on the use of psychoactive substances apart from alcohol
4. whether any unintended impacts of MUP, such as changes to alcohol sources and psychoactive substance use differ across Age group, Gender, Highest educational attainment, and Employment status
5. whether any observed intervention effects vary at the second and third time points

Study design

A repeated cross-sectional survey of patients attending SHCs was conducted to determine changes in drinking patterns and psychoactive substance use within this population. All patients attending three SHCs in Scotland and three in North England were invited to self-complete a short questionnaire. Data collection took place over three weeks at baseline, and eight and 12 months post-baseline. Timing was similar to the ED audit.

Study population

Patients of any age attending sexual health services participating in the study during data collection periods were approached. Patients who are unable to understand English well enough to complete the questionnaire (with assistance), or who left the clinic before an approach was made, and those deemed inappropriate by clinical staff to approach were excluded from the study.

Data collection

For peer review only - http://bmjopen.bmj.com/site/about/guidelines.xhtml

The questionnaire was offered to attendees at reception by a trained reception staff. Participants completed the questionnaire while waiting to be seen and returned it into a confidential returns box. The trained staff were available to offer assistance where needed. The confidential and voluntary nature of the study was emphasised. Similar patient completed questionnaires have been carried out as part of NHS Board work within NHS Lothian and NHS Greater Glasgow and Clyde achieving response rates of between 50-70% (without the assistance of support staff).

Questions covered where alcohol was procured, other substances used, the FAST tool (allowing quantification of alcohol misuse and binge drinkers), and basic characteristics (age band, sex, highest educational level). The questionnaire was designed to allow completion within between two and five minutes.

Socioeconomic position and inequalities

Trained reception staff assisted self-completion if required where literacy problems or visual impairment otherwise precluded participation. Analysis will be undertaken by highest education level, age and sex to determine if behavioural response to MUP in terms of drinking, source of alcohol and displacement is differentially patterned.

Outcome measures

The primary outcome from C2, which aims to reveal unintended effects, will be the proportion of patients self-reporting psychoactive substance use other than alcohol in the last month. Secondary outcomes will be drug-specific rates of use within the last month, sources of alcohol purchase (including cross-border, internet, smuggled or moonshine), rates of higher risk drinking (as measured by FAST score $\geq$3), self-reported binge-drinking, and analyses for differential displacement of use of illicit psychoactive drugs (including age, gender, highest achieved education, problematic drinkers)

Power calculation

We envisaged being able to recruit at least 50% of attendees at sexual health clinics at the six sites (three Scotland, three England), therefore resulting in a total of slightly over 10,000 recruits over two 3 week periods (an average of 288 per clinic per week). This would give us power of more than 80% to detect a change of $\pm$ 4% from an estimated 30% in the proportion of people using drugs.

Statistical analysis

Similar analyses to the ED component will be undertaken based on regression models with site included as a fixed effect, and the proportion taking illicit psychoactive drugs as the dependent variable for the primary outcome. We will examine whether any displacement effects are sustained between six and 12 months post implementation. We will investigate interactions with defined important covariates (including age, gender, highest achieved education, problematic drinkers) to investigate the possibility of differential intervention harms.

Ethical arrangements

For peer review only

To protect patient confidentiality, no identifiable personal data was collected. A research assistant was available to provide a questionnaire, answer any questions and assist those experiencing difficulty with self-completion. Given that young people are a particular focus of this study, no lower age restriction was planned. No separate written consent was planned as completion of the questionnaire was considered to constitute implied consent.

Component 3: Qualitative study of young people & heavy drinkers

The aim of this study component is to provide an in-depth understanding of how MUP is affecting key sub groups within the Scottish population. Understanding the experience of those exposed to the intervention allowed further exploration of potential mechanisms that result in unanticipated benefits and harms and which may affect groups to different degrees. This study contributes specifically to Research Aims two and three by exploring participants' expectations and experiences of the policy's impact including unintended consequences, both personally and on family, friends and wider community, and by comparing narratives between the different sample groups: age, gender and socioeconomic position.

Research design and data collection

Focus groups and interviews were carried out in January to April 2018 and repeated in September/October 2018 in three communities in Scotland; an affluent urban community, and two deprived urban communities. All data were collected by a mixed gender team of four full time researchers at the University of Stirling with extensive experience of qualitative methods. All participants were residents or professional stakeholders located within the catchment areas served by the two Scottish hospitals participating in the ED study component. Postcode deprivation (Carstairs) scores were used to define study communities in each area which matched the socioeconomic comparators of interest (deprived and affluent). Those who expressed an interest in the study were provided a copy of the study information sheet and an opportunity to ask questions before consenting to take part. Discussion and face-to-face interviews were conducted in local community venues or stakeholders place of work in private spaces to maintain confidentiality. Separate interview schedules and topic guides were devised for each study group and revised between waves.

1) Focus groups were conducted with those sub-groups who may be particularly affected by MUP: young people and heavy drinkers (previously categorised using the AUDIT C tool, a brief 3-item alcohol screening tool designed to identify hazardous and harmful drinking patterns) living in both deprived and affluent communities. Group participants were recruited by independent market researchers using door-to-door and street intercept techniques and were not known to the researchers. Recruitment was facilitated by the use of a recruitment questionnaire incorporating AUDIT-C screening questions. Each discussion covered social norms and attitudes, alcohol displacement behaviours, and changing patterns in drinking and purchasing habits.

For peer review only - http://bmjopen.bmj.com/site/about/guidelines.xhtml

2) Semi-structured interviews with professional stakeholders positioned to observe immediate social, health and economic impacts of MUP were conducted at each wave. These participants were not known by the study team prior to the study commencing. As well as addressing the main research objectives, data from these interviews were used to characterise the implementation process within each study community from differing professional perspectives and to explore adequacy of implementation and any difficulties experienced. In addition, these data provided valuable contextual information for informing the focus group topic guides and identifying lines of enquiry specific to each study community.

Study population

The focus groups recruited young and heavy drinkers (assessed as above) in three contrasting study communities: an affluent urban community, and two deprived urban communities in Scotland. Focus group participants were recruited purposively (as described above), for the two population groups of interest, young binge drinkers aged 18-25 years and older heavy drinkers aged 30 years and over. At each wave 12 single-sex focus groups were planned with an average of five to six participants in each group. The number and composition of the groups were selected to enable comparisons across two key study dimensions: types of drinker and socioeconomic status; and to facilitate open and free discussion allowing each participant the time to express their views.

Semi-structured interviews were conducted at each wave with key stakeholders working within each of the three study communities. The professional groups from which the stakeholder sample was drawn included representatives from licensing bodies, police, youth services, general practice and alcohol and drug treatment services. Stakeholders were identified using cascading techniques with a ceiling of 15-18 interviews at each wave.

Analysis plan

Focus group discussions and interviews were audio recorded and transcribed in full with participants' consent for analysis. Transcriptions were coded thematically using Nvivo12 software to allow the sharing of data across the research team and to enable identification of typical quotes, common reasoning and deviant or contradictory cases. Common themes and sub-themes were identified across the two data sets (drinkers and stakeholders) to assess for continuity of findings and to provide a more vivid and rounded picture of community expectations, the implementation process and the legislation's short-term impact. Together it is anticipated that these data will help to illuminate the findings to emerge from other study components.

<u>Patient and public involvement</u>

The development of the research question and outcome measures was informed by patients' priorities, experiences and preferences in two ways: through a review of media representations of MUP as a proxy source of information[62], survey research on patient

For peer review only - http://bmjopen.bmj.com/site/about/guidelines.xhtml

perceptions around alcohol consumption and price[81] and focus group and other research on public attitudes to MUP[64-67].

Patients were not directly involved in the design of the study, but were interviewees and respondents, and focus groups included members of the public. Service users with lived experiences of alcohol-related harms were represented on the study steering committee. Results will be disseminated to the public by actively engaging the mass media, using plain language summaries, a website, and posters at sites.

Dissemination plan

Given the policy relevance of this work, dissemination will be targeted to academic, policy and public audiences using tailored outputs.

Academic papers will include:

1. EDs study: the impact of minimum unit pricing on alcohol-related attendances at EDs
2. SHCs study: changes in intoxicating substance use amongst young people attending SHCs following minimum unit pricing
3. Qualitative study: public expectations and understandings of minimum unit pricing, and changes in social experiences of alcohol use following its introduction

Further papers may be produced, dependent on the timing of data collection and following investigation for population subgroup-specific effects.

A series of short policy briefings and presentations will be produced to summarise both quantitative and qualitative findings. We will produce an overall theoretical interpretation utilising all three components (see table 1) in the final study report.

These will be primarily aimed at policymakers within the Scottish and UK Governments, for example the Department of Health, Public Health England, Public Health Wales, the Public Health Agency in Northern Ireland, and NHS Health Scotland, but will be disseminated more broadly, subject to demand. The work will also be included in the review report NHS Health Scotland will produce for the Scottish Parliament and Ministers. This will provide evidence for Ministers' and the Scottish Parliament's decision about whether to renew the MUP legislation or allow it to fall under the 'sunset clause'. The academic and health and social care policy communities will be targeted through conference presentations.

Public audiences will be engaged through the production of plain-language summaries. These will be made available through a dedicated study website (http://sphsu.mrc.ac.uk/mup) which research participants will be signposted to (via the participant information leaflet). Posters summarising key results will be made available to study sites, so that findings of the research can be seen by both staff and patients. Broader public engagement will be achieved through an active mass media communication strategy, supported by ancillary use of social media when appropriate.

Data storage

For peer review only - http://bmjopen.bmj.com/site/about/guidelines.xhtml

Except as required for analysis, we have ensured that the electronic questionnaire data, consent forms, and data linkage forms are stored separately in three secure databases to maintain full confidentiality for these data.

Any paper-based data collected for the EDs study are stored, de-identified, in locked storage areas with strictly controlled access and handled according to MRC Good Research Practice Guidelines. Consents to participation in the study and to data linkage are stored separately in a dedicated secured area. Similarly, paper consent and data linkage forms are stored separately in a dedicated secured area.

SHCs data contain no personal identifiers, and are stored securely in a locked storage room and, when entered electronically, in a secure database.

Data Management

The original data were imported for data management and the original files backed up unaltered on a network drive only accessible to the project team. Data cleaning, manipulations and analysis were performed in separate coding/syntax files. We will provide well documented (i.e. with extensive commenting) syntax to ensure that all processing of data and results are reproducible.

Ethics review determined study integrity to be dependent upon retaining the anonymity of study participants and areas, including comparator sites. The protected period will apply from the start of the study to 1st September 2030.

Storage and use of data after the end of the study

Long term storage of data is at Iron Mountain, a commercial data storage facility. All data will be held in secure conditions with references for the contents of the sealed boxes held on a database for paper-held data. For electronic data secure electronic archives are used. Access to the data is controlled and permission from the data controller for the study is required to access long-term archived data. The electronic data will be archived at Glasgow University and destroyed by 1 September 2030 (ten years after the end of the study grant) as stated in R&D applications to research sites.

Strict data protection policies will be followed as outlined in the University of Glasgow's data protection policy.[82] The data will be worked from and stored upon a secure protected server (only accessible to the project team). Upon completion of the project, the data will then be archived in line with University of Glasgow University guidance on data archiving and the MRC's 'Personal Information in Medical Research' guidance document.

Data Statement

Data will be held by the University of Glasgow Social and Public Health Sciences Unit.

Acknowledgements

For peer review only - http://bmjopen.bmj.com/site/about/guidelines.xhtml

PD is supported by South London and Maudsley (SLaM) NHS Foundation Trust and by the National Institute for Health Research (NIHR) Biomedical Research Centre (BRC) for Mental Health at King's College London and SLaM. CD is supported by the NIHR Collaborations for Leadership in Applied Health Research South London at King's College Hospital Foundation Trust, the NIHR BRC for Mental Health at King's College London and SLaM, and is in receipt of an NIHR Senior Investigator Award. TP is supported by the NIHR Clinical Research Network for Yorkshire and The Humber. The views expressed here are those of the authors and do not necessarily reflect the views of the Department of Health and Social Care or NIHR.

The iPad application (app) used for data collection in EDs was developed by Codeface Ltd. The authors would like to thank NHS colleagues at all the research sites, who cannot be named for confidentiality reasons, for their assistance with this work:

The authors also wish to thank Julie Breslin and Kenneth Crawford of Addaction, who contributed a non-statutory and service user perspective to the Study Steering Committee.

In addition, we are particularly grateful to the Population Health Research Facility, and its predecessor, the Social and Public Health Sciences Surveys Unit at the University of Glasgow for their help with non-salary project costing, study training set up, study documents design, study operations set up, data entry, and data management.

For peer review only

Author Contributions

SVK, LB and CB conceived the study. SVK, CB, CD, DE, LG, SH, AL, GM, MS, LB and AHL all contributed to the overall study design and grant application. LB provided initial strategic leadership for the study, followed by AHL. TP and PD provided additional input on the design and conduct of study component 1 (Emergency Department component). AHL provided statistical expertise for designing study components 1 and 2. AF provided additional input on the design and conduct of study component 3 (Qualitative component). ADM and RF led the study day to day, contributed to parts of the methodology used, helped acquire and analyse the data. SVK wrote the first draft of the protocol, which was substantially revised and finalised by ADM. All authors made substantial contributions to study development, critically revised the paper and approved the final manuscript.

Funding

This study was funded by the NIHR Public Health Research Programme (11/3005/40).

The Social and Public Health Sciences Unit is funded by the Medical Research Council (MC_UU_12017/13 & MC_UU_12017/15) and Scottish Government Chief Scientist Office (SPHSU13 & SPHSU15). SVK acknowledges funding from a NRS Senior Clinical Fellowship (SCAF/15/02).

For peer review only - http://bmjopen.bmj.com/site/about/guidelines.xhtml

Department of Health and Social Care disclaimer

The views expressed are those of the authors and not necessarily those of the NHS, the NIHR or the Department of Health and Social Care.

Competing interests

CB, GM and LG are members of the Scottish-Government funded Monitoring and Evaluating Scotland's Alcohol Strategy (MESAS) evaluation. The remaining authors declare that they have no conflicts of interest.

Ethics approval

Ethical approval has been obtained from the NHS through the Scotland A Research Ethics Committee for study components 1 and 2, REC references are 12/SS/0120 and 12/SS/0121.

The ethics approval for component 3 was obtained in 2012 from the University of Stirling Management School ethics Committee (Application numbers 32 and 33).

For peer review only

References

1. Burgess L. Alcohol (Minimum Pricing) (Scotland) Bill: Passage of the Bill. Edinburgh, 2012.
2. Scottish Parliament. Alcohol (Minimum Pricing) (Scotland) Act 2012 (asp 4). Edinburgh: The Stationary Office Limited, 2012.
3. Katikireddi SV, Hilton S, Bonell C, et al. Understanding the Development of Minimum Unit Pricing of Alcohol in Scotland: A Qualitative Study of the Policy Process. *PLoS ONE* 2014;9(3):e91185. doi: 10.1371/journal.pone.0091185
4. Katikireddi SV, McLean JA. Introducing a minimum unit price for alcohol in Scotland: considerations under European Law and the implications for European public health. *The European Journal of Public Health* 2012;22(4):457-58. doi: 10.1093/eurpub/cks091
5. Katikireddi S V, Rice P, Holmes J, et al. Lessons from Scotland's experience of minimum unit pricing of alcohol. In: Giesbrecht N, Bosma L M, eds. Preventing Alcohol-Related Problems : Evidence and Community-Based Initiatives. Washington DC: American Public Health Association 2017.
6. Beeston C, Robinson M, Craig N, et al. Monitoring and Evaluating Scotland's Alcohol Strategy. Setting the Scene: Theory of change and baseline picture. Edinburgh: NHS health Scotland, 2011.
7. Abajobir AA, Abate KH, Abbafati C, et al. Global, regional, and national comparative risk assessment of 84 behavioural, environmental and occupational, and metabolic risks or clusters of risks, 1990-2016: a systematic analysis for the Global Burden of Disease Study 2016. *The Lancet* 2017;390(10100):1345-422. doi: 10.1016/S0140-6736(17)32366-8
8. Leon DA, McCambridge J. Liver cirrhosis mortality rates in Britain from 1950 to 2002: an analysis of routine data. *Lancet (London, England)* 2006;367(9504):52-6. doi: 10.1016/s0140-6736(06)67924-5 [published Online First: 2006/01/10]
9. Tod E, Grant I, Wyper G, et al. Hospital admissions, deaths and overall burden of disease attributable to alcohol consumption in Scotland. Glasgow: Scottish Public Health Observatory/NHS Health Scotland, 2018.
10. Casswell S, Thamarangsi T. Reducing harm from alcohol: call to action. *The Lancet* 2009;373(9682):2247-57.
11. Beaglehole R, Bonita R. Alcohol: a global health priority. *The Lancet* 2009;373(9682):2173-74.
12. Gilmore I. Action needed to tackle a global drink problem. *The Lancet* 2009;373(9682):2174-76.
13. Room R, Babor T, Rehm J. Alcohol and public health. *The Lancet* 2005;365(9458):519-30.
14. World Health Organisation. Global status report on alcohol and health Luxembourg, 2014.
15. Holder HD. Alcohol and the Community: A Systems Approach to Prevention. Cambridge: Cambridge University Press 1998.
16. Babor T, Caetano R, Casswell S, et al. Alcohol: No ordinary commodity: Research and public policy. 2nd ed. Oxford: Oxford University Press 2010.
17. Herttua K, Mäkelä P, Martikainen P. Differential trends in alcohol-related mortality: a register-based follow-up study in Finland in 1987–2003. *Alcohol and Alcoholism* 2007;42(5):456-64. doi: 10.1093/alcalc/agl099
18. Mäkelä P, Valkonen T, Martelin T. Contribution of deaths related to alcohol use to socioeconomic variation in mortality: register based follow up study. *BMJ* 1997;315(7102):211-16. doi: 10.1136/bmj.315.7102.211
19. Leyland A, Dundas R, McLoone P, et al. Cause-specific inequalities in mortality in Scotland: two decades of change. A population-based study. *BMC Public Health* 2007;7(1):172.
20. Mackenbach JP, Kulhánová I, Bopp M, et al. Inequalities in Alcohol-Related Mortality in 17 European Countries: A Retrospective Analysis of Mortality Registers. *PLoS Med* 2015;12(12):e1001909. doi: 10.1371/journal.pmed.1001909
21. Meier PS, Holmes J, Angus C, et al. Estimated Effects of Different Alcohol Taxation and Price Policies on Health Inequalities: A Mathematical Modelling Study. *PLoS Med* 2016;13(2):e1001963. doi: 10.1371/journal.pmed.1001963

For peer review only - http://bmjopen.bmj.com/site/about/guidelines.xhtml

MUP protocol paper v3 27.03.19

22. Holmes J, Meng Y, Meier PS, et al. Effects of minimum unit pricing for alcohol on different income and socioeconomic groups: a modelling study. *The Lancet* 2014;383(9929):1655-64. doi: http://dx.doi.org/10.1016/S0140-6736(13)62417-4
23. Katikireddi SV, Whitley E, Lewsey J, et al. Socioeconomic status as an effect modifier of alcohol consumption and harm: analysis of linked cohort data. *The Lancet Public Health* 2017;2(6):e267-e76. doi: 10.1016/S2468-2667(17)30078-6
24. Booth A, Meier P, Stockwell T, et al. Independent Review of the Effects of Alcohol Pricing and Promotion - Part A: Systematic Reviews. Sheffield: ScHARR, University of Sheffield, 2008.
25. Wagenaar AC, Salois MJ, Komro KA. Effects of beverage alcohol price and tax levels on drinking: a meta-analysis of 1003 estimates from 112 studies. *Addiction* 2009;104(2):179-90. doi: 10.1111/j.1360-0443.2008.02438.x
26. Huaung CD. Econometric models of alcohol demand in the UK. London: Government Economic Service, 2003.
27. Wagenaar AC, Tobler AL, Komro KA. Effects of Alcohol Tax and Price Policies on Morbidity and Mortality: A Systematic Review. *American Journal of Public Health* 2010;100(11):2270-78. doi: 10.2105/AJPH.2009.186007
28. Elder RW, Lawrence B, Ferguson A, et al. The Effectiveness of Tax Policy Interventions for Reducing Excessive Alcohol Consumption and Related Harms. *American Journal of Preventive Medicine* 2010;38(2):217-29. doi: 10.1016/j.amepre.2009.11.005
29. World Health Organization. Global strategy to reduce the harmful use of alcohol. Geneva, 2010.
30. Chisholm D, Moro D, Bertram M, et al. Are the “Best Buys” for Alcohol Control Still Valid? An Update on the Comparative Cost-Effectiveness of Alcohol Control Strategies at the Global Level. *Journal of Studies on Alcohol and Drugs* 2018;79(4):514-22. doi: 10.15288/jsad.2018.79.514
31. Burton R, Henn C, Lavoie D, et al. A rapid evidence review of the effectiveness and cost-effectiveness of alcohol control policies: an English perspective. *The Lancet* 2017;389(10078):1558-80. doi: https://doi.org/10.1016/S0140-6736(16)32420-5
32. Babor T, Caetano R, Casswell S, et al. Controlling affordability: Pricing and taxation. Alcohol: No ordinary commodity: Research and public policy. Oxford: Oxford University Press 2010:109-26.
33. UK Government. Tax on shopping and services 2018 [Available from: https://www.gov.uk/tax-on-shopping/alcohol-tobacco accessed 15/11/2018.
34. House of Commons Health Committee. Alcohol: First Report of Session 2009–10. London: House of Commons, 2009.
35. Health and Sport Committee. 2nd Report, 2012 (Session 4): Stage 1 Report on the Alcohol (Minimum Pricing) (Scotland) Bill. Edinburgh: Scottish Parliament, 2012.
36. Black H, Gill J, Chick J. The price of a drink: levels of consumption and price paid per unit of alcohol by Edinburgh's ill drinkers with a comparison to wider alcohol sales in Scotland. *Addiction* 2011;106(4):729-36. doi: 10.1111/j.1360-0443.2010.03225.x
37. Crawford MJ, Parry AMH, Weston ARW, et al. Relationship Between Price Paid for Off-Trade Alcohol, Alcohol Consumption and Income in England: A Cross-Sectional Survey. *Alcohol and Alcoholism* 2012;47(6):738-42. doi: 10.1093/alcalc/ags091
38. Purshouse RC, Meier PS, Brennan A, et al. Estimated effect of alcohol pricing policies on health and health economic outcomes in England: an epidemiological model. *The Lancet* 2010;375(9723):1355-64. doi: doi: 10.1016/S0140-6736(10)60058-X
39. Meng Y, Purshouse R, Brennan A, et al. Model-based Appraisal of Alcohol Minimum Pricing and Off-Licensed trade discount bans in Scotland using the Sheffield Alcohol Policy Model (v 2): An update based on newly available data. Sheffield: Scharr, University of Sheffield, 2010.
40. Angus C, Holmes J, Pryce R, et al. Model-based appraisal of the comparative impact of Minimum Unit Pricing and taxation policies in Scotland. Sheffield: University of Sheffield, 2016.

For peer review only

41. Bennetts R. IAS Briefing Paper: Use of Alcohol as a Loss-Leader. St Ives: Institute of Alcohol Studies, 2008.
42. Stockwell T, Leng J, Sturge J. Alcohol Pricing and Public Health in Canada: Issues and Opportunities. British Columbia: Centre for Addictions Research of BC, 2006.
43. Thompson K, Stockwell T, Wettlaufer A, et al. Minimum alcohol pricing policies in practice: A critical examination of implementation in Canada. *Journal of Public Health Policy* 2017;38(1):39-57. doi: 10.1057/s41271-016-0051-y
44. Katikireddi SV, Smith KE. Minimum unit pricing of alcohol in Scotland. In: Hill M, ed. Studying Public Policy. Bristol: Policy Press 2014.
45. Stockwell T, Zhao J, Giesbrecht N, et al. The raising of minimum alcohol prices in Saskatchewan, Canada: impacts on consumption and implications for public health. *Am J Public Health* 2012;102(12):e103-10. doi: 10.2105/AJPH.2012.301094 [doi] [published Online First: 2012/10/20]
46. Stockwell T, Zhao J, Martin G, et al. Minimum Alcohol Prices and Outlet Densities in British Columbia, Canada: Estimated Impacts on Alcohol-Attributable Hospital Admissions. *American Journal of Public Health* 2013:e1-e7. doi: 10.2105/ajph.2013.301289
47. Stockwell T, Auld MC, Zhao J, et al. Does minimum pricing reduce alcohol consumption? The experience of a Canadian province. *Addiction* 2012;107(5):912-20. doi: 10.1111/j.1360-0443.2011.03763.x
48. Stockwell T, Zhao J, Sherk A, et al. Assessing the impacts of Saskatchewan's minimum alcohol pricing regulations on alcohol-related crime. *Drug and Alcohol Review* 2016:n/a-n/a. doi: 10.1111/dar.12471
49. Zhao J, Stockwell T. The impacts of minimum alcohol pricing on alcohol attributable morbidity in regions of British Colombia, Canada with low, medium and high mean family income. *Addiction* 2017;112(11):1942-51. doi: doi:10.1111/add.13902
50. Stockwell T, Zhao J, Marzell M, et al. Relationships Between Minimum Alcohol Pricing and Crime During the Partial Privatization of a Canadian Government Alcohol Monopoly. *Journal of Studies on Alcohol and Drugs* 2015;76(4):628-34. doi: 10.15288/jsad.2015.76.628
51. Sherk A, Stockwell T, Callaghan RC. The effect on emergency department visits of raised alcohol minimum prices in Saskatchewan, Canada. *Drug and Alcohol Review* 2018;37(S1) doi: doi:10.1111/dar.12670 [published Online First: 12/02/2018]
52. McKee M. Alcohol in Russia. *Alcohol and Alcoholism* 1999;34(6):824-29. doi: 10.1093/alcalc/34.6.824
53. Holden C, Hawkins B. 'Whisky gloss': The alcohol industry, devolution and policy communities in Scotland. *Public Policy and Administration* 2012 doi: 10.1177/0952076712452290
54. Katikireddi SV, Bond L, Hilton S. Changing Policy Framing as a Deliberate Strategy for Public Health Advocacy: A Qualitative Policy Case Study of Minimum Unit Pricing of Alcohol. *Milbank Quarterly* 2014;92(2):250-83.
55. Hawkins B, Holden C. Framing the alcohol policy debate: industry actors and the regulation of the UK beverage alcohol market. *Critical Policy Studies* 2013;7(1):53-71. doi: 10.1080/19460171.2013.766023
56. McCambridge J, Hawkins B, Holden C. Industry Use of Evidence to Influence Alcohol Policy: A Case Study of Submissions to the 2008 Scottish Government Consultation. *PLoS Medicine* 2013;10(4):e1001431. doi: 10.1371/journal.pmed.1001431
57. Hawkins B, Holden C. 'Water dripping on stone'? Industry lobbying and UK alcohol policy. *Policy & Politics* 2014;42(1):55-70. doi: 10.1332/030557312x655468
58. Katikireddi SV, Bond L, Hilton S. Perspectives on Econometric Modelling to Inform Policy: A UK Qualitative Case Study of Minimum Unit Pricing of Alcohol. *The European Journal of Public Health* 2014;24(3):490-95. doi: doi:10.1093/eurpub/ckt206

For peer review only - http://bmjopen.bmj.com/site/about/guidelines.xhtml

59. Katikireddi SV, Hilton S, Bond L. The role of the Sheffield model on the minimum unit pricing of alcohol debate: the importance of a rhetorical perspective. *Evidence and Policy: A Journal of Research, Debate and Practice* 2016;12(4):521-39.
60. Wood K, Patterson C, Katikireddi SV, et al. Harms to 'others' from alcohol consumption in the minimum unit pricing policy debate: a qualitative content analysis of UK newspapers (2005–12). *Addiction* 2014;109:578-84. doi: 10.1111/add.12427
61. Katikireddi SV, Hilton S. How did policy actors use mass media to influence the Scottish alcohol minimum unit pricing debate? Comparative analysis of newspapers, evidence submissions and interviews. *Drugs: Education, Prevention, and Policy* 2014;22(2):125-34. doi: 10.3109/09687637.2014.977228
62. Patterson C, Katikireddi SV, Wood K, et al. Representations of minimum unit pricing for alcohol in UK newspapers: a case study of a public health policy debate. *Journal of Public Health* 2015;37(1):40-49.
63. Hilton S, Wood K, Patterson C, et al. Implications for alcohol minimum unit pricing advocacy: What can we learn for public health from UK newsprint coverage of key claim-makers in the policy debate? *Social Science & Medicine* 2014;102(0):157-64. doi: http://dx.doi.org/10.1016/j.socscimed.2013.11.041
64. Lonsdale A, Hardcastle S, Hagger M. A minimum price per unit of alcohol: A focus group study to investigate public opinion concerning UK government proposals to introduce new price controls to curb alcohol consumption. *BMC Public Health* 2012;12(1):1023.
65. Pechey R, Burge P, Mentzakis E, et al. Public acceptability of population-level interventions to reduce alcohol consumption: A discrete choice experiment. *Social Science and Medicine* 2014;113(0):104-09. doi: http://dx.doi.org/10.1016/j.socscimed.2014.05.010
66. Li J, Lovatt M, Eadie D, et al. Public attitudes towards alcohol control policies in Scotland and England: Results from a mixed-methods study. *Social Science & Medicine* 2017;177(Supplement C):177-89. doi: https://doi.org/10.1016/j.socscimed.2017.01.037
67. O'May F, Gill J, Black H, et al. Heavy Drinkers' Perspectives on Minimum Unit Pricing for Alcohol in Scotland:A Qualitative Interview Study. *SAGE Open* 2016;6(3):2158244016657141. doi: 10.1177/2158244016657141
68. Craig P, Katikireddi SV, Leyland AH, et al. Natural experiments: An overview of methods, approaches and contribution to public health intervention research. *Annual Review of Public Health* 2017;38(1):39-56.
69. Craig P, Cooper C, Gunnell D, et al. Using natural experiments to evaluate population health interventions: new Medical Research Council guidance. *JECH* 2012;66:1182-6. doi: 10.1136/jech-2011-200375
70. NHS Health Scotland. Monitoring and Evaluating Scotland's Alcohol Strategy (MESAS): Briefing paper. December 2009 Glasgow, 2009.
71. Bonell C, Jamal F, Melendez-Torres GJ, et al. 'Dark logic': theorising the harmful consequences of public health interventions. *Journal of Epidemiology and Community Health* 2014 doi: 10.1136/jech-2014-204671
72. Hodgson R, Alwyn T, John B, et al. THE FAST ALCOHOL SCREENING TEST. *Alcohol and Alcoholism* 2002;37(1):61-66. doi: 10.1093/alcalc/37.1.61
73. National Institute for Health and Care Effectiveness N. Alcohol-use disorders: prevention. Public Health Guideline (PH24), 2010.
74. Saunders JB, Aasland OG, Babor TF, et al. Development of the Alcohol Use Disorders Identification Test (AUDIT): WHO Collaborative Project on Early Detection of Persons with Harmful Alcohol Consumption--II. *Addiction* 1993;88(6):791-804. [published Online First: 1993/06/01]
75. Hodgson RJ, John B, Abbasi T, et al. Fast screening for alcohol misuse. *Addictive behaviors* 2003;28(8):1453-63. [published Online First: 2003/09/27]

For peer review only - http://bmjopen.bmj.com/site/about/guidelines.xhtml

76. Jones LA. Systematic review of alcohol screening tools for use in the emergency department. *Emergency medicine journal : EMJ* 2011;28(3):182-91. doi: 10.1136/emj.2009.085324 [published Online First: 2010/10/16]
77. Phillips T. What is the burden of alcohol related problems on accident and emergency departments (AED) in England?: An epidemiological analysis of the prevalence of alcohol use disorders (AUDs) within the AED setting. King's College, 2016.
78. Drummond D, Deluca P, Coulton S, et al. The Effectiveness of Alcohol Screening and Brief Intervention in Emergency Departments: A Multicentre Pragmatic Cluster Randomized Controlled Trial. . *PLoS ONE* 2014;9(6):e99463. doi: https://doi.org/10.1371/journal.pone.0099463
79. Drummond D, Phillips T, Coulton S, et al. Saturday Night and Sunday Morning: the 2003 twenty-four hour national survey of alcohol-related attendances at accident and emergency departments in England. Final report to the Cabinet Office. . London: Institute of Psychiatry, 2003.
80. NHS Quality Improvement Scotland. NHS Quality Improvement Scotland. Scottish Emergency Department Alcohol Audit: Understanding Alcohol Misuse in Scotland – Final Report. Edinburgh, 2008.
81. Cook PA, Phillips-Howard PA, Morleo M, et al. The Big Drink Debate: perceptions of the impact of price on alcohol consumption from a large scale cross-sectional convenience survey in north west England. *BMC public health* 2011;11:664-64. doi: 10.1186/1471-2458-11-664
82. University of Glasgow, Data Protection Policy 2006 [Available from: http://www.gla.ac.uk/services/dpfoioffice/policiesandprocedures/dpa-policy/.

For peer review only

For peer review only - http://bmjopen.bmj.com/site/about/guidelines.xhtml

STROBE 2007 (v4) Statement—Checklist of items that should be included in reports of *cross-sectional studies*

Section/Topic	Item #	Recommendation	Reported on page #
**Title and abstract**	1	(*a*) Indicate the study's design with a commonly used term in the title or the abstract	Title p1
		(*b*) Provide in the abstract an informative and balanced summary of what was done and what was found	Abstract p2
**Introduction**			
Background/rationale	2	Explain the scientific background and rationale for the investigation being reported	Pp4-6
Objectives	3	State specific objectives, including any prespecified hypotheses	EDs Pp7-8,<br>SHCs p11,<br>Qualitative pp12-13
**Methods**			
Study design	4	Present key elements of study design early in the paper	EDs/SHCs P5,<br>Qualitative p13
Setting	5	Describe the setting, locations, and relevant dates, including periods of recruitment, exposure, follow-up, and data collection	EDs Pp7-9,<br>SHCs p11-12,<br>Qualitative p13
Participants	6	(*a*) Give the eligibility criteria, and the sources and methods of selection of participants	EDs P8,<br>SHCs p11,<br>Qualitative P13
Variables	7	Clearly define all outcomes, exposures, predictors, potential confounders, and effect modifiers. Give diagnostic criteria, if applicable	EDs Pp9-10,<br>SHCs p14,
Data sources/ measurement	8*	For each variable of interest, give sources of data and details of methods of assessment (measurement). Describe comparability of assessment methods if there is more than one group	EDs p9<br>SHCs pp11-12<br>Qualitative pp13-14
Bias	9	Describe any efforts to address potential sources of bias	EDs pp9-10<br>SHCs p12<br>Qualitative p14
Study size	10	Explain how the study size was arrived at	EDs p10<br>SHCs p12

For peer review only

			Qualitative p13
Quantitative variables	11	Explain how quantitative variables were handled in the analyses. If applicable, describe which groupings were chosen and why	EDs p10<br>SHCs p12<br>Qualitative n/a
Statistical methods	12	(*a*) Describe all statistical methods, including those used to control for confounding	EDs p10<br>SHCs p12<br>Qualitative n/a
		(*b*) Describe any methods used to examine subgroups and interactions	EDs p10<br>SHCs p12<br>Qualitative n/a
		(*c*) Explain how missing data were addressed ***NOT applicable as this is a protocol paper***	EDs<br>SHCs<br>Qualitative
		(*d*) If applicable, describe analytical methods taking account of sampling strategy ***NOT applicable from here on as this is a protocol paper***	EDs<br>SHCs<br>Qualitative
		(*e*) Describe any sensitivity analyses ***NOT applicable from here on as this is a protocol paper***	EDs<br>SHCs<br>Qualitative
**Results**		***NOT applicable from here on as this is a protocol paper***	
Participants	13*	(a) Report numbers of individuals at each stage of study—eg numbers potentially eligible, examined for eligibility, confirmed eligible, included in the study, completing follow-up, and analysed	EDs<br>SHCs<br>Qualitative
		(b) Give reasons for non-participation at each stage	EDs<br>SHCs<br>Qualitative
		(c) Consider use of a flow diagram	EDs<br>SHCs<br>Qualitative
Descriptive data	14*	(a) Give characteristics of study participants (eg demographic, clinical, social) and information on exposures and potential confounders	EDs<br>SHCs

For peer review only

			Qualitative
		(b) Indicate number of participants with missing data for each variable of interest	EDs<br>SHCs<br>Qualitative
Outcome data	15*	Report numbers of outcome events or summary measures	EDs<br>SHCs<br>Qualitative
Main results	16	(*a*) Give unadjusted estimates and, if applicable, confounder-adjusted estimates and their precision (eg, 95% confidence interval). Make clear which confounders were adjusted for and why they were included	EDs<br>SHCs<br>Qualitative
		(*b*) Report category boundaries when continuous variables were categorized	EDs<br>SHCs<br>Qualitative
		(*c*) If relevant, consider translating estimates of relative risk into absolute risk for a meaningful time period	EDs<br>SHCs<br>Qualitative
Other analyses	17	Report other analyses done—eg analyses of subgroups and interactions, and sensitivity analyses	EDs<br>SHCs<br>Qualitative
**Discussion**			
Key results	18	Summarise key results with reference to study objectives	EDs<br>SHCs<br>Qualitative
Limitations	19	Discuss limitations of the study, taking into account sources of potential bias or imprecision. Discuss both direction and magnitude of any potential bias	EDs<br>SHCs<br>Qualitative
Interpretation	20	Give a cautious overall interpretation of results considering objectives, limitations, multiplicity of analyses, results from similar studies, and other relevant evidence	EDs<br>SHCs<br>Qualitative
Generalisability	21	Discuss the generalisability (external validity) of the study results	EDs<br>SHCs

For peer review only

			Qualitative
**Other information**			
Funding	22	Give the source of funding and the role of the funders for the present study and, if applicable, for the original study on which the present article is based	EDs/SHCs/Qualitative P17

*Give information separately for cases and controls in case-control studies and, if applicable, for exposed and unexposed groups in cohort and cross-sectional studies.

Note: An Explanation and Elaboration article discusses each checklist item and gives methodological background and published examples of transparent reporting. The STROBE checklist is best used in conjunction with this article (freely available on the Web sites of PLoS Medicine at http://www.plosmedicine.org/, Annals of Internal Medicine at http://www.annals.org/, and Epidemiology at http://www.epidem.com/). Information on the STROBE Initiative is available at www.strobe-statement.org.

For peer review only

Manuscript: **Evaluating possible intended and unintended consequences of the implementation of alcohol minimum unit pricing (MUP) in Scotland: a natural experiment protocol**

Consolidated criteria for reporting qualitative studies (COREQ): 32-item checklist

Developed from:
Tong A, Sainsbury P, Craig J. Consolidated criteria for reporting qualitative research (COREQ): a 32-item checklist for interviews and focus groups. *International Journal for Quality in Health Care*. 2007. Volume 19, Number 6: pp. 349 – 357

No. Item	Guide questions/description	Reported on Page #
**Domain 1: Research team and reflexivity**		
Personal Characteristics		
1. Interviewer/facilitator	Which author/s conducted the inter view or focus group?	Page 13
2. Credentials	What were the researcher's credentials? E.g. PhD, MD	Page 13
3. Occupation	What was their occupation at the time of the study?	Page 13
4. Gender	Was the researcher male or female?	Page 13
5. Experience and training	What experience or training did the researcher have?	Page 13
Relationship with participants		
6. Relationship established	Was a relationship established prior to study commencement?	Page 14 .
7. Participant knowledge of the interviewer	What did the participants know about the researcher? e.g. personal goals, reasons for doing the research	Page 14
8. Interviewer characteristics	What characteristics were reported about the interviewer/facilitator? e.g. Bias, assumptions, reasons and interests in the research topic	Page 13 implicit in full-time researchers' role

For peer review only

**Domain 2: study design**		
Theoretical framework		
9. Methodological orientation and Theory	What methodological orientation was stated to underpin the study? e.g. grounded theory, discourse analysis, ethnography, phenomenology, content analysis	Page 14 implicit
Participant selection		
10. Sampling	How were participants selected? e.g. purposive, convenience, consecutive, snowball	Pages 13 and 14
11. Method of approach	How were participants approached? e.g. face-to-face, telephone, mail, email	Pages 13-14
12. Sample size	How many participants were in the study?	Page 14
13. Non-participation	How many people refused to participate or dropped out? Reasons?	Not covered, will be in main paper
Setting		
14. Setting of data collection	Where was the data collected? e.g. home, clinic, workplace	Pages 13-14 .
15. Presence of non-participants	Was anyone else present besides the participants and researchers?	Page 14
16. Description of sample	What are the important characteristics of the sample? e.g. demographic data, date	Page 14
Data collection		
17. Interview guide	Were questions, prompts, guides provided by the authors? Was it pilot tested?	Page 13
18. Repeat interviews	Were repeat inter views carried out? If yes, how many?	Page 13
19. Audio/visual recording	Did the research use audio or visual recording to collect the data?	Page 14
20. Field notes	Were field notes made during and/or after the inter view or focus group?	Page 14 (implicit)
21. Duration	What was the duration of the interviews or focus group?	Not specified, will be reported in main

For peer review only

		results paper
22. Data saturation	Was data saturation discussed?	Page 13-14 not specified
23. Transcripts returned	Were transcripts returned to participants for comment and/or correction?	Page 13-14 not specified
**Domain 3: analysis and findings**		
Data analysis		
24. Number of data coders	How many data coders coded the data?	N/A for protocol paper, but at least two
25. Description of the coding tree	Did authors provide a description of the coding tree?	N/A for protocol paper
26. Derivation of themes	Were themes identified in advance or derived from the data?	N/A for protocol paper (will be derived from the data)
27. Software	What software, if applicable, was used to manage the data?	N/A for protocol paper
28. Participant checking	Did participants provide feedback on the findings?	N/A for protocol paper
Reporting		
29. Quotations presented	Were participant quotations presented to illustrate the themes/findings? Was each quotation identified? e.g. participant number	N/A for protocol paper, but quotations will be used in results paper
30. Data and findings consistent	Was there consistency between the data presented and the findings?	N/A for protocol paper
31. Clarity of major themes	Were major themes clearly presented in the findings?	N/A for protocol paper
32. Clarity of minor themes	Is there a description of diverse cases or discussion of minor themes?	N/A for protocol paper

For peer review only
